# Computational mechanisms underlying latent value updating of unchosen actions

**DOI:** 10.1126/sciadv.adi2704

**Published:** 2023-10-20

**Authors:** Ido Ben-Artzi, Yoav Kessler, Bruno Nicenboim, Nitzan Shahar

**Affiliations:** ^1^ School of Psychological Sciences, Tel Aviv University, Tel Aviv, Israel.; ^2^ Sagol School of Neuroscience, Tel Aviv University, Tel Aviv, Israel.; ^3^ Minducate Science of Learning Research and Innovation Center of the Sagol School of Neuroscience, Tel Aviv University, Tel Aviv, Israel.; ^4^ Department of Psychology and School of Brain Sciences and Cognition, Ben Gurion University of the Negev, Be'er Sheva, Israel.; ^5^ Department of Cognitive Science and Artificial Intelligence, Tilburg University, Tilburg, Netherlands.

## Abstract

Current studies suggest that individuals estimate the value of their choices based on observed feedback. Here, we ask whether individuals also update the value of their unchosen actions, even when the associated feedback remains unknown. One hundred seventy-eight individuals completed a multi-armed bandit task, making choices to gain rewards. We found robust evidence suggesting latent value updating of unchosen actions based on the chosen action’s outcome. Computational modeling results suggested that this effect is mainly explained by a value updating mechanism whereby individuals integrate the outcome history for choosing an option with that of rejecting the alternative. Properties of the deliberation (i.e., duration/difficulty) did not moderate the latent value updating of unchosen actions, suggesting that memory traces generated during deliberation might take a smaller role in this specific phenomenon than previously thought. We discuss the mechanisms facilitating credit assignment to unchosen actions and their implications for human decision-making.

## Teaser

Humans internally update the value of options not chosen even in the absence of counterfactual feedback.

## INTRODUCTION

Humans are known to make choices according to the expected value of available actions. A wealth of research has suggested that humans learn action values through an iterative process of trial and error, whereby the value of each action is updated according to observable and temporally adjacent outcomes (*1*). However, for many decisions, the outcome of actions we deliberated upon but did not commit to remains hidden. For example, we might deliberate on which form of transportation is best to commute to our first day at work, by bus or by train. If we choose to take the bus, the train experience remains unavailable to us, thus limiting our ability to update its value. Reinforcement learning models have extensively examined how humans update action values according to observed outcomes. However, a fundamental question remains regarding whether and how humans may falsely update the value of unchosen actions for which no feedback was observed.

Previous studies suggested that the co-occurrence of the chosen and unchosen actions during deliberation forms a unique memory association due to the shared context (*2*–*4*). Accordingly, an unchosen action may linger in one’s mind even after a decision has been made due to memory traces generated during the deliberation (*5*–*7*). Memory research has demonstrated the co-occurrence of value updating in associated cognitive representations (*8*–*11*). Studies suggest that when memory binds two representations together (e.g., in classical conditioning), a value update of one representation induces an update of the associated second representation (*12*–*15*). Therefore, shared episodic memory for the chosen and unchosen actions could lead to corresponding value updates during feedback presentation.

Current research has further argued that instead of a direct value updating, unchosen actions may be updated inversely to the chosen action’s feedback [i.e., if a chosen action is rewarded, the value of the unchosen action is reduced; (*16*, *17*)]. According to this idea, the context of the deliberation creates a negative association in one’s mind between the chosen and unchosen options. The notion here is that a reactivation of the chosen item during value updating should also reactivate the deliberation context where the individual “teased apart” and contrasted the value of the two options. Therefore, reactivation of the deliberation context should lead to inverse, rather than matching, value update for unchosen actions according to the observed outcome. Another reason to assume that the value of the unchosen action should be inversely updated is that when making a choice, the act of committing to an option is always confounded with a decision to reject the alternative. Therefore, any value assigned to a chosen action might also reinforce the decision to avoid the alternative. For example, if taking the bus rather than the train turns out to lead to the desired outcome, the individual might learn not to take the train the next day even if deliberated against other forms of transportation.

In a recent pioneering study, Biderman and Shohamy (*17*) examined value updating for unchosen options in a single-shot decision paradigm and found inverse value updating of unchosen options according to the chosen option outcome. However, while this research provided a compelling first line of evidence, several key questions are left unanswered. First, it is still unknown whether inverse value updating for unchosen options will be observed in a sequential decision task where options are reoffered for selection multiple times and individuals learn long-run expected value for each alternative. Second, Biderman and Shohamy suggested that the unique context of the deliberation where the individual needs to tease apart the values of the alternatives leads to a negative and inverted memory association between the value representations of the offered options. This contrastive binding of options in memory is then assumed to lead to the unchosen action value being inversely updated following the feedback for the chosen action. Following this theoretical notion, we examined whether estimates that are well known as indicators for the deliberation process [i.e., reaction time (RT) and choice difficulty; (*18*)] moderate the extent of value updating for unchosen actions. Third, the exact latent computational mechanism according to which unchosen values are updated remains unknown.

**Fig. 1.**
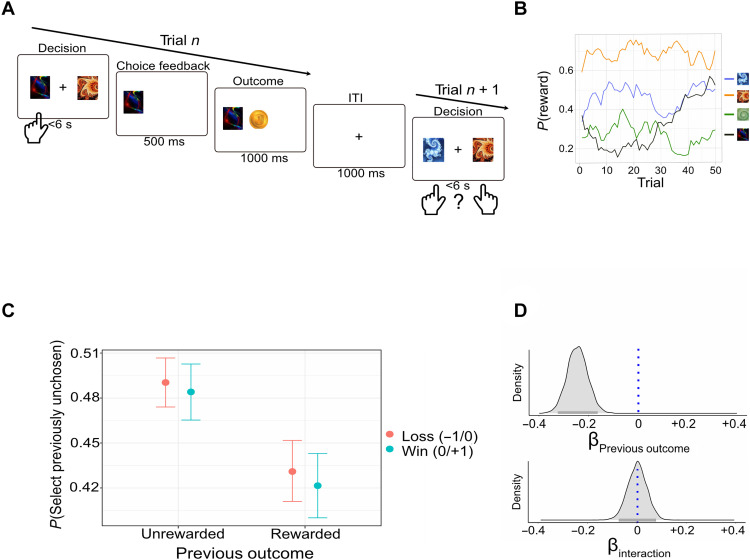
Inverse value updating of unchosen actions. (**A**) Illustration of a trial sequence. Participants completed a four-armed bandit task. In each trial, two cards (of four) were randomly offered by the computer for participants’ selection. We examined trials where the unchosen card in trial *n* was reoffered at trial *n* + 1 with a card that was not offered on trial *n*. This allowed us to examine whether the outcome associated with the chosen card in trial *n* influenced the probability that the participant will select the previously unchosen card at trial *n +* 1. For example, as illustrated in this panel, we ask whether the reward delivered at trial *n* (as a result of choosing the dark card) influenced the probability of selecting the unchosen card (orange) when offered with a third card (blue). (**B**) Card selection led to a binary outcome determined by slowly drifting probabilities. We used randomly drifting reward probabilities to ensure continued learning. The reward probabilities of each card were independent (mean shared variance = 5.3%). (**C**) Probability of choosing a previously unchosen action as a function of outcome in the previous trial. Results indicated that the probability of choosing a previously unchosen card was reduced after rewarded trials compared to unrewarded trials. This was true for both win blocks (where outcomes included winning/not winning a play pound coin) and loss blocks (where outcomes included not winning/losing a play pound coin). (**D**) The posterior distributions for the influence of previous outcome (top) and the interaction with condition (bottom) on choosing the previously unchosen card in a logistic regression (the blue dotted line indicates the null point, and the gray horizontal line indicates HDI_95%_). Overall results indicate an inverted influence of the previous outcome on the chances of selecting an unchosen action, regardless of win/loss conditions.

In the current study, we used a reinforcement learning paradigm together with computational modeling to explore the mechanisms underlying the value update of unchosen actions. Specifically, participants completed a multi-armed bandit task during which they were asked to make card selections to gain monetary rewards. In each trial, participants were offered two cards (randomly selected by the computer from a deck of four cards) and then were presented with the monetary outcome associated with the chosen card. The paradigm allowed us to tease apart the value of the unchosen from the chosen card by selectively analyzing trials in which a previously unchosen card was offered with a different third card that was not presented in the previous deliberation trial (see Fig. 1). The temporal adjacency of deliberation and outcome embedded in our design allowed us to test the extent to which the inverse value update of unchosen actions was dependent on the properties of the deliberation process. The use of adjacent and repeated choices and outcomes allowed us to further investigate, using computational modeling, the prediction-error mechanism underlying the value update of unchosen actions (*19*–*22*).

First, our results demonstrated latent credit assignment to unchosen actions in the context of a consecutive choice-outcome trial design. Contrary to our intuition, we found that properties

of the deliberation (i.e., duration and difficulty) did not moderate the extent of value updating for unchosen actions. Rather, reinforcement learning computational modeling suggested that for every action, individuals consider the outcome history for choosing an option, together with the outcome history of rejecting the alternative. This mechanism predicted the effect of value update for unchosen actions on both the group and individual levels. We discuss these findings in light of current theoretical considerations.

## RESULTS

We studied the choice behavior of 178 participants who completed a multi-armed bandit reinforcement learning task online. In this task, participants were asked to choose cards to gain monetary rewards. In each trial, participants were randomly offered two of four possible cards by the computer. After choosing a card, participants could be rewarded or not according to a drifting probability. Half the task included winning blocks (rewarded versus unrewarded outcomes were +£1/0, respectively), and half included loss blocks (rewarded versus unrewarded outcomes were £0/−1, respectively). We were interested in examining whether participants assigned credit (i.e., value) to cards that were considered, but unchosen, compared to cards that were not considered during deliberation. We start by reporting and replicating model-independent sequential trial analyses, allowing us to demonstrate inverse credit assignment to unchosen actions. Next, we examine to what extent properties of the deliberation moderated latent value update of unchosen actions. Last, we use computational reinforcement learning modeling to examine possible internal updating mechanisms for unchosen actions.

### Model-independent analysis

#### Accuracy rates

As a first step, to ensure that participants were able to adequately perform the task, we examined overall accuracy rates. Accurate choices were defined as choices where the individual chose the card with the higher true latent expected values. We found an above-chance accuracy rate for both loss [56.8% accuracy, 95% equal-tailed density interval (HDI_95%_) between 55.5 and 58.0] and win blocks (58.1% accuracy, HDI_95%_ between 57.0 and 59.2). Accuracy rates improved with trial progression (see fig. S1 for learning curves) and increased for easier trials where the difference between the true latent expected values of the two cards was higher (see fig. S2). Therefore, overall participants learned to act according to the true expected values of the cards and were able to choose the better card above chance.

#### Influence of reward on unchosen actions

To address the main aim of the study, we performed a consecutive trial analysis allowing us to examine whether participants assigned credit to an unchosen card based on the outcome of the chosen card. Specifically, we examined only trials where the unchosen card from trial *n* was reoffered on trial *n* + 1. We further filtered and took only trials where the previously chosen card was not reoffered (see Fig. 1A). We performed a hierarchical Bayesian logistic regression where previous outcome for the chosen card in trial *n* (rewarded versus unrewarded) predicted the tendency to choose in trial *n* + 1 a card that was unchosen in trial *n.* We found strong evidence for the influence of previous outcome on participants’ choices such that participants were less likely to choose a previously unchosen card in trial *n* + 1 if the chosen card in trial *n* was rewarded (43%) versus unrewarded (49%; Fig. 1, B and C; posterior median = −0.25, HDI_95%_ = −0.33 to −0.17; probability of direction = ~100%; see the Supplementary Materials for prior robustness checks). To confirm the robustness of this empirical finding, we performed three additional complementary analyses:

##### Sensitivity to loss versus win conditions

The effect of previous outcome was similar for both win (7.3%) and loss blocks (6.8%; see Fig. 1C). Specifically, we repeated the same regression analysis with the addition of block type (win versus loss) and block type × previous outcome as additional predictors. We found no evidence for an interaction effect with block type such that participants were similarly likely to be affected by the previous outcome regardless of block type (interaction posterior median = −0.0005, HDI_95%_ between −0.08 and 0.07; probability of direction = 50.6%).

##### Replication with emphasized instructions

We repeated the same analysis with a second existing dataset from our laboratory where participants completed a similar four-armed bandit task online (*N* = 49; see the Supplementary Materials for further information) (*23*). The instruction phase in this second dataset emphasized and quizzed participants to ensure that they understood that monetary outcomes reflect the value of the chosen card alone and that whether a certain card is more/less valuable in a given trial had no relation to whether other cards are more or less valuable. This instruction was objectively correct, since in our design the true expected values of the arms were independent. Despite that, we found the same effect suggesting that participants tended to choose the previously unchosen option less often in trial *n* + 1 if trial *n* was rewarded (45%) versus unrewarded (51%; posterior median = −0.19, HDI_95%_ between −0.32 and −0.06; probability of direction = 99.8%).

##### Extending the effect to two trials back

We repeated the same analysis, only with respect to three consecutive trials (influence of outcome in trial *n*, on probability to select the unchosen option at trial *n* + 2), and found a similar yet somewhat smaller effect showing that participants were less likely to pick the previously unchosen card in trial *n* + 2 if trial *n* was rewarded (44%) versus unrewarded (46%; posterior median = −0.10, HDI_95%_ between −0.17 and −0.02; probability of direction = 99%).

#### Moderation of deliberation on value updating of unchosen actions

Previous studies suggested that the context of the deliberation binds in memory the two alternatives (*17*, *24*). Therefore, we aimed to extend our main finding (Fig. 1C) and examine whether the magnitude of latent value update for unchosen actions is moderated by the deliberation process. We operationalized the deliberation process by two indicators, RT and choice difficulty, both commonly used in decision-making literature. Specifically, studies have suggested that RTs encapsulate decision time (*25*, *26*). Accordingly, a prolonged deliberation process was shown to be indicated by slower RTs (*27*, *28*). Furthermore, it is well established that more difficult choices (where the accurate or best alternative is not easily distinct) lead to a prolonged deliberation process (*18*, *29*–*32*). Studies have also shown that longer decision times are associated with stronger memory associations (*33*). Following that motivation, we performed the following two analyses.

##### (i) Interaction with decision time

**Fig. 2.**
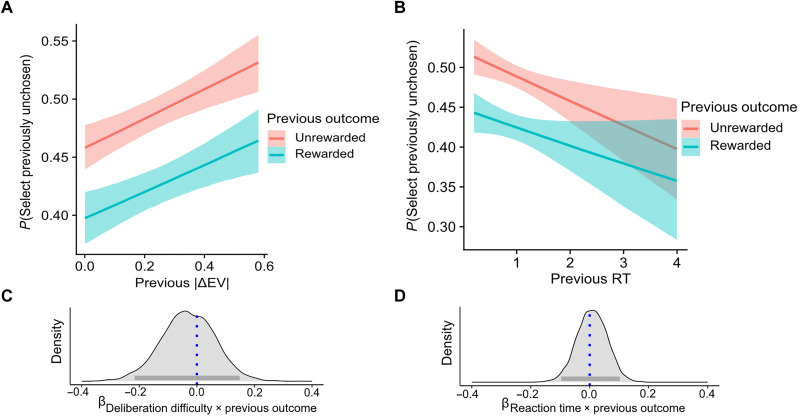
Moderation of deliberation duration and difficulty on inverse value updating of unchosen actions. (**A**) Hierarchical Bayesian logistic regression showed no moderating effect for the absolute difference in expected values between the two offered cards on the tendency to assign value to an unchosen action. (**B**) Higher RTs were assumed to be indicative of increased deliberation but had no moderation effect on the tendency to assign value for unchosen actions. (**C**) Posterior distribution showing no evidence supporting the moderation of value updating for unchosen actions by deliberation difficulty (blue line indicating the null point; probability of direction = 65%; gray line indicating HDI_95%_). (**D**) Posterior distribution depicting a lack of evidence for the interaction between RT and previous outcome on the tendency to choose a previously unselected card (blue line indicating the null point; probability of direction = 55%; gray line indicates HDI_95%_).

We hypothesized that a longer decision time will lead to increased memory associations between the two cards and will increase the inverse value updating of unchosen cards based on the outcome of the chosen card. Therefore, we examined whether RT in the previous trial moderated latent value update for unchosen actions. We performed a logistic regression with previous outcome, previous RT, and their paired interaction as predictors for choosing in trial *n +* 1 the card that was previously unchosen at trial *n* (see Fig. 1). As in the previous analysis, this was done only for trials where the chosen card was not reoffered. We found no evidence for a moderating effect of previous RT such that participants were similarly likely to be affected by the previous outcome on trial *n* in choosing a previously unchosen card in trial *n* + 1 regardless of their RT (Fig. 2B; interaction posterior median = 0.008, HDI_95%_ = −0.09 to 0.11; probability of direction = 55%).

##### (ii) Interaction with decision difficulty

We hypothesized that a more difficult deliberation process would lead to a stronger inverted memory association between the chosen and unchosen options (*16*, *17*). To estimate deliberation difficulty, we calculated the absolute difference in true reward expected values between the two offered cards. We reasoned that a smaller difference in true expected value between the two offered cards should reflect a more difficult deliberation process. Therefore, we examined whether decision difficulty, defined as the absolute difference between the expected value of the two offers in the previous trial, moderated value learning for unchosen actions. We performed a logistic regression with previous outcome, previous difficulty, and their paired interaction. We found no evidence for a moderating effect of previous difficulty (i.e., the absolute difference in expected values on the previous trial) on the tendency to assign value to unchosen actions. Specifically, participants were similarly likely to be affected by the previous outcome on trial *n* in choosing a previously unchosen card in trial *n* + 1 regardless of the true expected value difference (Fig. 2A; interaction posterior median = −0.03, HDI_95%_ = −0.21 to 0.15; probability of direction = 65%). We repeated the same analysis, only with difficulty of the current trial as additional predictor to ensure that the update of unchosen actions is robust and independent of the true running average of the four arms. We found the effect of the previous reward on choosing the previously unchosen option to be independent of the difference in expected values of the currently suggested options (interaction posterior median = 0.0003, HDI_95%_ = −0.3 to 0.3; probability of direction = 50%; see fig. S3). Overall, we found that the value assignment to unchosen actions was independent of the previous and current trial difficulty. Thus, we found no evidence in favor of a moderating role of deliberation duration or difficulty on the inverse value update of unchosen action.

Together, model-independent analyses showed clear evidence both in our main dataset and in a further replication in favor of an inverse and latent update of unchosen actions based on the outcome associated with the chosen card. We found no evidence that deliberation difficulty and duration (assumed to induce memory associations between chosen and unchosen cards) moderated the inverse value update of unchosen actions. Our findings, suggesting a lack of association between latent update of unchosen actions and deliberation properties, may only be applicable to the specific experimental setup used in this study. In particular, our observations pertain to sequential decision-making tasks where feedback is provided instantaneously. We now turn to computational modeling using Q-learning algorithms to further examine value update mechanisms that might underlie such an effect.

#### Computational modeling

To explore the updating process of the unchosen action, we fit several reinforcement learning models to participants’ choice behavior. In all models, we updated the chosen action according to a well-established prediction-error mechanism where the difference between the expected and the observed outcome is used as a value teaching signal (*1*, *34*, *35*). However, the models differed in their theoretical assumptions regarding the inversion of information used to update the value of unchosen actions.

1) Double updating with two prediction errors: Here we assumed that the participant holds the belief that the arms are anticorrelated and generates internally an outcome for the unchosen action in an inverse direction to the external outcome. Therefore, in the current model, we included a separate prediction error for the unchosen action that is calculated as the difference between an inverted “hallucinatory” outcome and the expected value of the unchosen action [Eq. 5; (*36*)].

2) Double updating with one prediction error: Here, we also assumed that participants might hold a belief regarding anticorrelations between the options. However, we relaxed the assumption that participants generate an internal hallucinatory outcome for the unchosen action. Instead, we assumed that the participant experiences only a single prediction error based on the difference between the external outcome and the expected value of the chosen action. Participants were further assumed to update the unchosen action based on an inverted portion of the prediction error of the chosen action (Eq. 7).

3) Select-reject mechanism: Here, we relaxed the assumption that the participant performs any inversion at all (as suggested by the two previous models). Instead, we assumed that the individual holds and updates in mind two separate values for selecting and rejecting each card. In the select-reject model, both select and reject values are updated according to separate prediction-error signals, and choices are determined by a weighted integration of the value of selecting a card with the value of rejecting the alternative (Eq. 12).

**Fig. 3.**
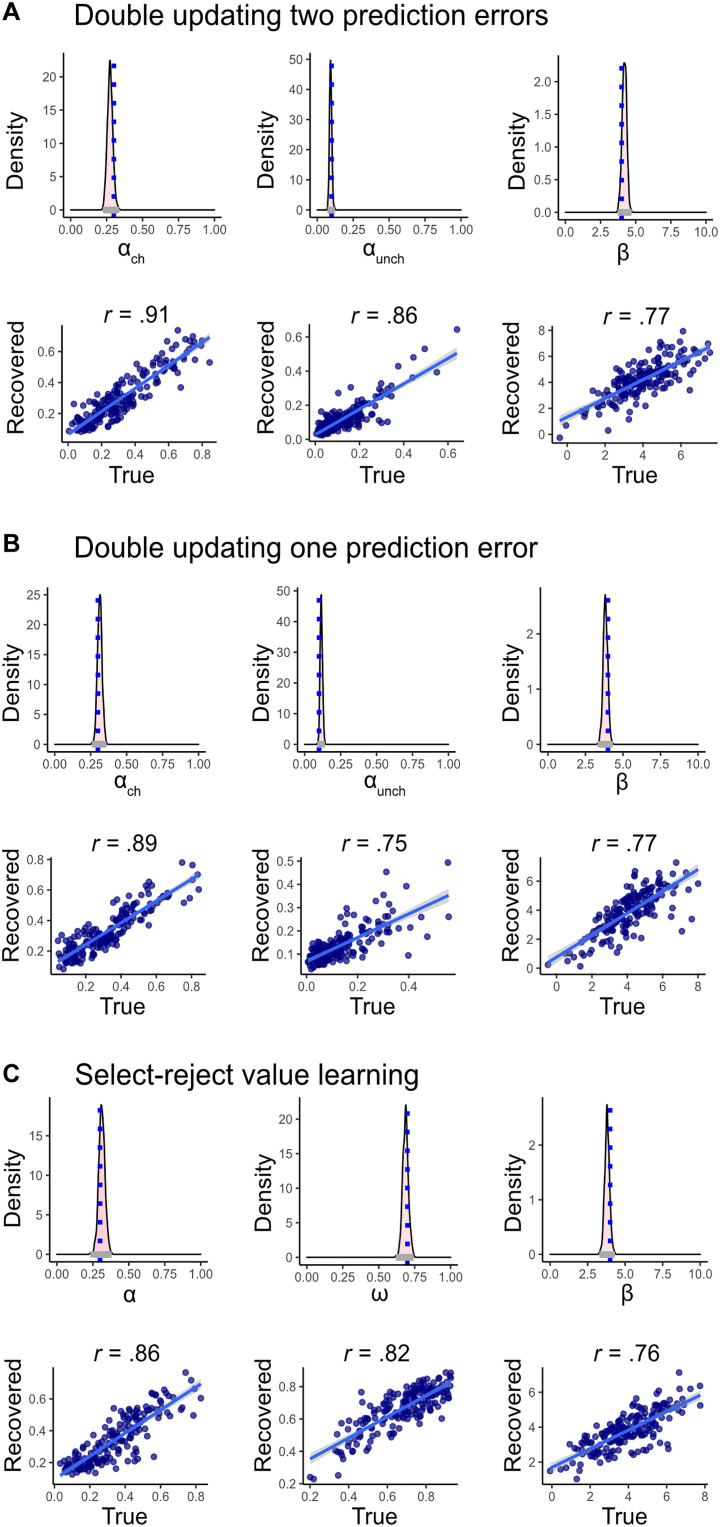
Parameter recovery. The top rows in each panel present population parameter recovery, including the posterior parameter distribution (pink) and the blue dashed line indicating the value of the true latent population parameter. The bottom rows refer to individual parameter recovery, showing a strong correlation between simulated individual parameters and recovered ones. (**A**) The three parameters of the “double updating with two prediction errors” model, (**B**) the “double updating with one prediction error” model, and (**C**) the “select-reject” model. Overall, we found good parameter recovery for all parameters and models.

**Fig. 4.**
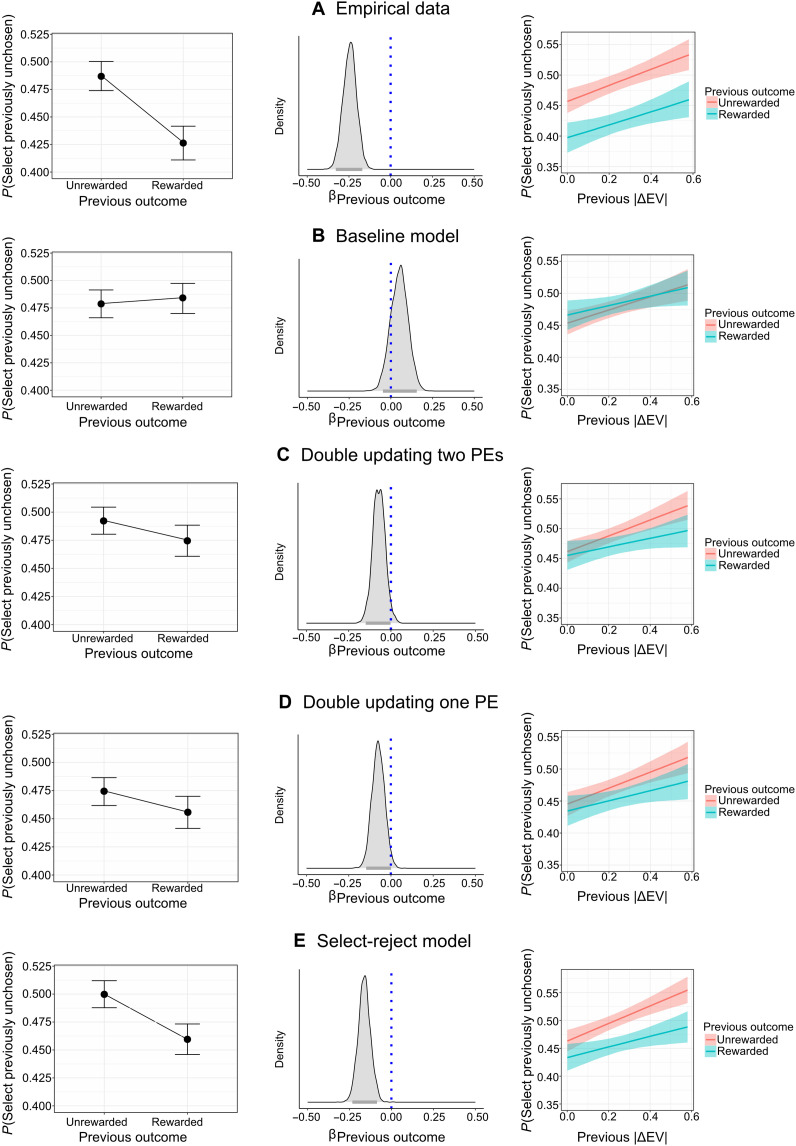
Simulated effects for computational models. (**A**) The main regression signatures found in the empirical data. For each model (**B** to **E**), we simulated data from 175 agents using empirical parameters (sampled from the population marginal posterior distributions). We then used the simulated data to examine the ability of each model to reproduce the main regression signatures we found in the empirical data. (Left and middle columns) The effect of previous outcome on the probability of choosing a previously unchosen action and the corresponding posterior distribution (estimates are presented on the log-odds scale). (Right column) The moderation of choice difficulty in the previous trial (indicated by absolute difference between the expected values of the two offers) on the effect of previous outcome on the probability of choosing a previously unchosen action. Overall, we found that the baseline model was unable to reproduce the effect of latent updating for unchosen actions. All other models were able to produce this effect in the same direction as the empirical data, with the select-reject model showing the closest effect to the empirical one. PE, prediction error.

The three suggested models had three parameters each and were compared to a classical baseline model in which only the value of the chosen card, but not the value of the unchosen card, was updated. Before testing and comparing the models with empirical data, a few tests were performed to ensure that model space is well defined and can provide meaningful results. First, we found excellent parameter recovery properties for all models (see Materials and Methods and Fig. 3). Second, we performed a model recovery analysis, testing our ability to identify the true data-generating model from observed data, in silico. We found excellent recoverability of all models (see Materials and Methods and Table 1). Last, we added a fourth baseline model for comparison purposes, in which the unchosen actions were not updated. We then simulated data from all models based on estimated parameters, examined, and found that the findings reported in Fig. 1C cannot be reproduced by the baseline model (see Fig. 4 and Materials and Methods). However, as expected, we found that all other models of interest were able to produce the inverse credit assignment signature. The models and parameters were highly recoverable, suggesting the adequacy of this computational design in investigating the mechanism underlying latent updating for unchosen actions. Overall, these measures ensure that our model space is well defined and can be recovered with confidence. We will now describe each model in detail and report our empirical model comparison results.

**Table 1.** Model recovery results. Values represent Δelpd referring to the difference between the elpd of the data-generating model (columns) and the elpd of the alternative (rows). elpd estimates were obtained using leave-one-block-out cross-validation for all models including the data-generating models. Negative values reflect a worse fit of the alternative model compared to the data-generating model estimates. Values in brackets represent the SE of the elpd difference distribution. An Δelpd difference that is more than twice the SE should be seen as substantial (*40*, *83*).

		Data-generating model		
		Select-reject	Double-updating with one prediction error	Double-updating with two prediction errors
Fitted model	Select-reject	0	−434 (28.9)	−725.8 (34.3)
	Double updating with one prediction error	−258.6 (22.2)	0	−671 (35.2)
	Double updating with two prediction errors	−150.3 (17.5)	−268.7 (23.3)	0
	Baseline	−591.6 (32.7)	−750.8 (39.1)	−1891.8 (56.4)

##### Baseline model

For each trial, we calculated a prediction-error teaching signal and updated action values (represented by *Q* values) using a temporal difference learning algorithm. The baseline model was a classical Rescorla-Wagner model, where only the chosen option was updated according to a reward prediction error

$$\delta_{\mathrm{chosen}}=(\mathrm{reward}-Q_{\mathrm{chosen}})$$ (1)

$$Q_{\mathrm{chosen}}=Q_{\mathrm{chosen}}+\alpha_{\mathrm{chosen}}\cdot \delta_{\mathrm{chosen}}$$ (2)

where α_chosen_ is a learning rate (free parameter) and δ_chosen_ indicates reward prediction error for the chosen option. *Q* values were used to predict each choice according to a softmax policy

$$p(\mathrm{choice})=\frac{\exp(\beta \cdot Q_{\mathrm{chosen}})}{\exp(\beta \cdot Q_{\mathrm{chosen}})+\exp(\beta \cdot Q_{\mathrm{unchosen}})}$$ (3)

where β is an inverse noise parameter (free parameter). Overall, the current model had two population-level free parameters (α_chosen_, β), along with their associated random effect parameters. To ensure that this model is unable to produce the observed logistic regression effect reported above, we repeated the same logistic regression analysis with simulated data. We found that this model was unable to produce the regression signature found in empirical data, which validated its use as a baseline model (see the Supplementary Materials and Fig. 4).

##### Model 1—Double updating with two prediction errors

Here, in addition to updating the chosen option, individuals were also assumed to experience a latent prediction error for the unchosen option (i.e., δ_unchosen_). We updated the unchosen option according to

$$\delta_{\mathrm{unchosen}}=[(1-\mathrm{reward})-Q_{\mathrm{unchosen}}]$$ (4)

$$Q_{\mathrm{unchosen}}=Q_{\mathrm{unchosen}}+\alpha_{\mathrm{unchosen}}\cdot \delta_{\mathrm{unchosen}}$$ (5)

where α_unchosen_ is a learning rate (free parameter) and δ_unchosen_ represents reward prediction error for the unchosen option. Note that δ_unchosen_ is calculated according to the difference between the inverted observed reward and the predicted value of the unchosen option. Chosen options were updated as in the baseline model (Eqs. 1 and 2), with each action predicted according to a softmax policy (Eq. 3). Overall, this model had the same two parameters as the baseline model, with an additional learning rate (i.e., α_unchosen_) and its associated random effect scale parameter.

##### Model 2—Double updating with one prediction error

Here, we assume that the individual experienced one prediction error based on the outcome and the predicted value of the chosen option (i.e., Eq. 1). However, unlike the baseline model, the current model assumes that a portion of this prediction error is inverted and assigned to the unchosen option. Therefore, here, we calculate a prediction error using Eq. 1 and then update the chosen and unchosen options according to

$$Q_{\mathrm{chosen}}=Q_{\mathrm{chosen}}+\alpha_{\mathrm{chosen}}\cdot \delta_{\mathrm{chosen}}$$ (6)

$$Q_{\mathrm{unchosen}}=Q_{\mathrm{unchosen}}+\alpha_{\mathrm{unchosen}}\cdot (-\delta_{\mathrm{chosen}})$$ (7)

where α_chosen_ and α_unchosen_ are the learning rates for chosen and unchosen options, respectively (e.g., when α_unchosen_ = 0, the model becomes exactly like the baseline model). Overall, this model had the same two parameters as the baseline model, with the addition of a learning rate parameter (i.e., α_unchosen_) and its associated random effect scale parameter.

##### Model 3—Select-reject value learning

Here, we assumed that the agent held in mind different values for when a card was selected or rejected. Therefore, the current model had a *Q*^select^ value and a *Q*^reject^ value for each possible action (a total of eight *Q* values for four possible cards in each block of the current task). An important aspect of this model is that it permits us to alleviate the assumption that participants engage in an additional process of mentally reversing the outcome before updating the value of the unselected option. The value of the chosen card was updated according to a reward prediction error

$$\delta_{\left(\mathrm{chosen}\right)}=[\mathrm{reward}-{Q^{\mathrm{select}}}_{\left(\mathrm{chosen}\right)}]$$ (8)

$${Q^{\mathrm{select}}}_{\left(\mathrm{chosen}\right)}={Q^{\mathrm{select}}}_{\left(\mathrm{chosen}\right)}+\alpha \cdot \delta_{\left(\mathrm{chosen}\right)}$$ (9)

The value of unchosen cards was updated according to

$$\delta_{\left(\mathrm{unchosen}\right)}=[\mathrm{reward}-{Q^{\mathrm{reject}}}_{\left(\mathrm{unchosen}\right)}]$$ (10)

$${Q^{\mathrm{reject}}}_{\left(\mathrm{unchosen}\right)}={Q^{\mathrm{reject}}}_{\left(\mathrm{unchosen}\right)}+\alpha \cdot \delta_{\left(\mathrm{unchosen}\right)}$$ (11)

Note that, here, the unchosen card is not updated according to an inverse reward signal, but rather the same reward value as the chosen action. Furthermore, the chosen and unchosen options were updated according to the same learning rate. Choices were predicted using an integrated *Q* value (i.e., *Q*^net^) and a softmax decision policy

$$\begin{array}{c} {Q^{\mathrm{net}}}_{\left(\mathrm{offer}1\right)}=\omega \cdot {Q^{\mathrm{select}}}_{\left(\mathrm{offer}1\right)}+(1-\omega )\cdot {Q^{\mathrm{reject}}}_{\left(\mathrm{offer}2\right)}\\ {Q^{\mathrm{net}}}_{\left(\mathrm{offer}2\right)}=\omega \cdot {Q^{\mathrm{select}}}_{\left(\mathrm{offer}2\right)}+(1-\omega )\cdot {Q^{\mathrm{reject}}}_{\left(\mathrm{offer}1\right)} \end{array}$$ (12)

$$p(\mathrm{choice})=\frac{\exp[\beta \cdot Q_{\left(\mathrm{chosen}\right)}^{\mathrm{net}}]}{\exp[\beta \cdot Q_{\left(\mathrm{chosen}\right)}^{\mathrm{net}}]+\exp[\beta \cdot Q_{\left(\mathrm{unchosen}\right)}^{\mathrm{net}}]}$$ (13)

The ω parameter indicated each individual’s tendency to weigh the value of selecting a certain card with the value of rejecting the alternative. In the extreme case where ω = 1, the model converges to the baseline model, and when ω = 0, the agent will base choices purely on the outcome history of rejecting offered cards. Therefore, in the current model, we assume that the subject deliberates on an integrated option of taking a card while rejecting the other. This allowed us to relax the assumption that individuals inverted internally the reward that was assigned to the unchosen card as was described in both models 1 and 2. Note that while this model holds some resemblance to model 1 (double updating with two prediction errors), it also has a distinct feature of segregating the outcome history for chosen and unchosen options. For example, let us take an example where an individual deliberated cards A versus B and decided to take card A. Under model 1 (double updating with two prediction errors), the value of card A, and hence its prediction error, will be based on all the trials where it was either chosen or unchosen (to the extent that the learning rate permits; Eq. 5). However, in the current select-reject mechanism (model 3), the value and the associated prediction error of A (the chosen card) will be calculated solely based on the outcome history for when A was chosen, while the value of B and its associated prediction error will similarly be based on trials where B was unchosen. Overall, this model had the same two parameters as the baseline model, with the addition of an additional decision weight (i.e., ω) and its associated random effect scale parameter.

#### Examining each model’s ability to generate the main behavioral effects

Following recent guidelines for model selection in computational cognitive modeling (*37*, *38*), we wanted to examine whether our models can generate the main regression results found in the empirical data (Figs. 1C and 2A). We therefore simulated data for each model based on empirical parameters (see Materials and Methods) and calculated and illustrated the effect of previous outcome on the selection of unchosen actions (see Fig. 4). We found that the findings reported in Figs. 1C and 2A were not reproduced by the baseline model (see Materials and Methods and the Supplementary Materials). However, as expected, we found that all other models of interest were able to produce the inverse credit assignment signature (see Fig. 4). Given that we also found good model recoverability for these models, we continued to perform model comparison using leave-one-block-out Bayesian estimation.

#### Model comparison

All models were fitted to the data using hierarchical Bayesian modeling via “stan” Markov chain Monte Carlo (MCMC) sampling [(*39*); see Materials and Methods]. For purposes of model comparison, we performed a leave-one-block-out cross-validation analysis. Specifically, in each round of the cross-validation procedure, we omitted one block, calculated hierarchically posterior distributions for each individual across all parameters based on the remaining blocks, and used these posterior distributions to predict trial-by-trial actions for the left-out observations. This allowed us to gain expected log-predictive density (i.e., elpd) distributions for each observed empirical action across all models. We then followed current well-accepted guidelines for Bayesian model comparison and compared elpd for each model, allowing us to quantify the ability of each model to accurately predict data that were not used for training (*40*). We then calculated the difference elpd distribution between the models (*40*). Table 2 reports the mean and SD of the elpd difference distribution for each model compared with the winning select-reject model. According to current established guidelines, a mean difference twice larger than the SE should be considered as evidence in favor of the winning model (*40*, *41*). As can be seen in Table 2, this criterion was reached for the winning model, suggesting superiority in terms of choice predictions compared with the alternatives (see Table 2). To further demonstrate the association between the winning model and empirical data, we report here two additional analyses.

**Table 2.** Model comparison results. elpd refers to expected log-probability density estimates calculated using leave-one-block-out cross-validation (larger values indicate better fit; see Materials and Methods). elpd SE and elpd difference SE are mentioned in brackets. elpd difference larger than four and at least twice the SE can be considered as a heuristic to decide which is the winning model (*40*).

Model	Expected log-predictive density (elpd)	elpd difference compared to the select-reject value learning model (winning model)
Baseline model	−22,236.8 (55.8)	−114.8 (19.4)
Double updating with one prediction error	−22,195.2 (58.1)	−73.2 (16.3)
Double updating with two prediction errors	−22,157.2 (59.0)	−35.2 (16.1)
Select-reject value learning	−22,122.0 (57.9)	0 (0)

##### Replicating individual differences from simulated data that are based on empirical parameters

**Fig. 5.**
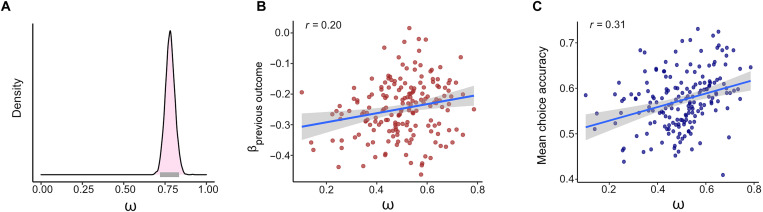
Results of the select-reject value learning model. (**A**) Population-level posterior distribution of the ω parameter in a hierarchical model. For ω = 1, individuals will consider the value of a card based on the reward history when that card was selected. For ω = 0, individuals will consider the value of a card only based on the reward history when the alternative was rejected. The posterior distribution suggests that participants weigh in their decision both the history of when a card was chosen and when the alternative was rejected, with greater emphasis on the former compared with the latter. The posterior high-density interval (gray horizontal line) is clearly below one, suggesting that individuals considered the value of actions not only based on trials where a card was chosen but also to a lesser degree based on trials where the alternative was rejected (i.e., 0.5 < ω < 1). (**B**) Association between median posterior ω parameter estimates for each individual and the model-independent effect estimated for each individual using empirical data (i.e., β_previous outcome_). The positive association demonstrates the model’s ability to capture individual differences. (**C**) Association between individuals’ ω parameter estimates and their mean choice accuracy. The correlation shows that higher ω values correlated with better performance accuracy in the current bandit task.

Next, we examined whether the winning select-reject value learning model also adequately captured individual differences in the influence of the chosen action’s feedback on the unchosen action value. Specifically, we examined the association between the ω parameter in the select-reject value learning model (weighting the contribution of reject value learning to decision-making) and individual estimates for the model-independent effect. We found a positive correlation (Pearson *r* = 0.20, BF_10_ = 7.54; probability of direction = 99.65; HDI_95%_ = 0.06 to 0.34) between the ω parameter and the individual’s previous-outcome coefficient (from the above logistic regression), showing that the parameter estimations of the computational and model-independent analysis are in line with each other (see Fig. 5B).

##### Choice accuracy and tendency to update unchosen actions

Last, we wanted to test accuracy rates in light of our main finding. In the current paradigm, the value of each card drifted independently, and knowledge about the value of one card was not predictive of the other. This means that updating the value of unchosen actions based on the outcome of a different (chosen) card should only reduce the accuracy of value estimation and lead to lower choice accuracy. However, we wanted to explore using empirical data whether inverse updating of unchosen actions did not lead to some unexpected monetary benefits, thus encouraging individuals to use such a strategy. We, therefore, estimated the association between individuals’ parameters in the winning model (model 3, select-reject) and individuals’ choice accuracy (defined as 1 if the participant selected the arm with the higher true expected value and 0 otherwise). We found a substantial positive correlation between ω and mean choice accuracy (Pearson *r* = 0.31; BF_10_ = 953.83; probability of direction = ~100%; see Fig. 5C). Therefore, if anything, updating unchosen actions was counterproductive and led to lower profits.

Overall, our computational model comparison results support the hypothesis of latent value updating of unchosen actions. We found clear evidence to suggest that the observed latent update of unchosen actions is less probable under a double-updating model (either with one or two prediction errors). The data seem more plausible under a select-reject model, where individuals are assumed to hold and update separate values in mind for rejecting and selecting an action. The primary rationale behind using the select-reject model is that it eliminates the need to assume that humans engage in a mental inversion of the received outcome to update the value of the unselected option. The winning model was able to reproduce the signature regression analysis examining the influence of previous outcome on the probability of selecting a previously unchosen action (see Fig. 4). The winning model was also able to explain individual differences in this effect (see Fig. 5, B and C).

## DISCUSSION

Prior studies have demonstrated counterfactual value-based learning in human choice behavior (*19*–*22*, *42*, *43*). However, these studies were conducted almost exclusively in the context of full feedback such that outcomes of both chosen and unchosen actions were observable. In the present study, we demonstrated latent value updating of unchosen actions in a multi-armed reinforcement learning task where no counterfactual feedback was available. This sequential design allowed us to explore the reward prediction-error mechanisms underlying latent value updating of unchosen actions and examine the extent to which properties of the deliberation process moderate such latent updating.

In the current study, we found that individuals assign value to unchosen actions based on the inverted observable value assigned to the chosen action; reward delivery in response to a chosen action reduced the likelihood of selecting a previously unchosen action, and loss increased its selection probability. Individuals were able to estimate the value of all four arms based on their choices, but unexpectedly, they still exhibited a tendency to update unchosen arms based on the outcomes of chosen arms. Since there was no dependency between the true expected values of the arms, the participants were not required, and had no benefit, in performing this counterfactual update. To the best of our knowledge, the only previous study that demonstrated an inverse value update for unchosen actions was a study by Biderman and Shohamy, which was recently further replicated (*17*, *24*). However, in their study, the deliberation and outcome phases were delivered in separate blocks. We extend their finding by showing similar inverse updating for unchosen actions in a standard reinforcement learning multi-armed bandit task in which outcomes immediately followed deliberation and choice. This pattern of findings demonstrated across different choice-outcome temporal variations attests to the robustness of the effect.

Our design allowed us to examine the influence of the deliberation process on the value updating of unchosen actions. A main theoretical explanation for the finding that individuals update unchosen actions according to the outcome of chosen actions is shared memory associations (*12*–*15*). Specifically, a prior study has argued that the deliberation process “ties” the chosen and unchosen actions in one’s mind such that a value update of one action leads to an update of the other (*17*). The unique context of the deliberation process, whereby individuals need to tease apart the value of two options, is assumed to further lead to an inverted, rather than direct, value update. The underlying theoretical assumption for this notion is that when the association between chosen and unchosen actions is reactivated during the value update process for the chosen action, the deliberation goal of teasing apart options based on their value is also in play, thus leading to an inverted value update of the unchosen action (*44*, *45*). Our findings showed no evidence in favor of a moderation effect of deliberation duration and difficulty on latent value updating of unchosen actions. Specifically, we found that neither RTs nor the difference in true expected value between the two options moderated the magnitude of the update for the unchosen action. Thus, it seems that although previous studies suggested that memory associations created during deliberation may lead to the effect at hand, this does not seem to be the case in this study. However, we cannot rule out the possibility that the sequential nature of our task, as well as the immediacy of the feedback, limits these findings to this specific paradigm. It is possible that since feedback in the current paradigm is abundant, participants tended to create fewer memory associations during deliberation, which led to our inability to find the aforementioned interaction. Furthermore, our analyses were merely correlative. Future studies may manipulate the deliberation process to search for further support for the claim that it has no impact on the degree of value update for unchosen options, for instance, by manipulating the speed-accuracy instructions (*46*) or by comparing a condition with value-based choices to arbitrary picking (*31*, *47*–*49*). Nevertheless, such manipulations should be carefully conducted as it is nontrivial to manipulate the deliberation process without interfering with its natural dynamics (*50*).

If properties of the deliberation process do not moderate inverse latent value updating of unchosen actions, what alternative mechanism may explain the latent and inverse value update? Computational modeling allowed us to explore three potential theoretical mechanisms, each including a different assumption regarding the inversion of information in mind during the update of unchosen actions; first, we assumed that the cognitive system might produce an internal outcome for the unchosen option (i.e., double updating with two prediction errors model). Here, the system is thought to generate a “hallucinated” outcome for the unchosen action that is perfectly anticorrelated with the chosen action’s outcome (*36*). This internal outcome is then assumed to lead to a second prediction error according to the difference between the inverted outcome and the unchosen option’s expected value. This idea is in line with previous neuroimaging studies indicating that separate neural populations might generate two independent prediction errors for a chosen and unchosen action (at least when full feedback is included) (*51*, *52*). Thus, we tested a double-updating model with two prediction errors assuming that participants produce an internal outcome for the unchosen option based on the evident outcome of the chosen option.

Second, we assumed that the cognitive system might process only a single outcome, without producing an internal outcome for the unchosen arm (i.e., double updating with one prediction error model). This suggests that only one “surprise” value (or prediction error) might be formed according to the external outcome and the anticipated value of the chosen action. This single prediction error is then assumed to be inverted internally to accommodate a belief individuals might hold regarding a negative correlation between choice values (*53*). This model allowed us to relax the assumption that the system generates internally a hypothetical outcome. Instead, under the current model, the system predicts a contrasting update for the unchosen option based on the surprise signal for the chosen action.

Last, we assumed that the cognitive system might not perform any inversion of information at all. Instead, it could maintain separate value representations for trials where an option was either selected or rejected (i.e., select-reject model). Like the two previous models, a select-reject cognitive mechanism can also lead to the result observed in Fig. 1C, as the value of rejecting an option increases when a rejection is rewarded (see Fig. 4). Our modeling results indicated evidence in favor of the latter mechanism (i.e., select-reject value learning model), whereby two independent prediction errors are calculated by the system for the arm that was selected and the one that was rejected.

According to the select-reject value learning model suggested by our results, an option’s value is determined as a combination of the previous outcomes in which it was chosen, and the alternative was rejected. An important theoretical benefit of the select-reject value learning model is that it allows us to relax the assumption that the system performed an inversion of values as suggested in the two double-updating models (*17*). It also does not require us to assume that the memory contrast between chosen and unchosen actions generated during deliberation is the mechanism driving the double and inverted update. Rather, we suggest that the two values, one for taking the chosen action and the other for rejecting the unchosen action, might linger in one’s mind until an outcome is observed, thus leading to a direct update of both values. We would like to assert that this theoretical suggestion does not imply that a negative memory association between chosen and unchosen actions does not take place (*8*, *11*, *13*, *15*), but rather we present a plausible alternative explanation that does not rely on memory associations that may arise during the deliberation process. However, one of the challenges of the current cognitive mechanism is that it also implies additional value representations to be stored in memory. To test this explanation, future studies can use dedicated sophisticated paradigms where only the value of rejecting alternatives can drive choices.

A relevant value update mechanism is choice-confirmation bias where individuals are suggested to show a larger value update for both chosen and unchosen actions following choice-confirming outcomes (*43*, *54*–*57*). Previous studies demonstrated that participants update the value of unchosen options when provided with counterfactual outcome feedback (also known as “full feedback” paradigms). Furthermore, several studies have indicated that chosen options receive a larger value update for positive versus negative outcomes. Palminteri and Lebreton (*56*) integrated these two findings and suggested a choice-confirmation bias mechanism. Here, participants are suggested to show a larger value update for both chosen options followed by positive obtained outcomes and unchosen options followed by negative foregone outcomes (in full feedback paradigms where the participants observe the outcome of both chosen and unchosen options). This mechanism effectively leads to an update of values in a confirmatory manner. In the current study, participants were provided with feedback for the chosen action alone without providing them with the foregone outcome. Extension of the computational modeling to include a choice-confirmatory bias showed, as expected, higher learning rates for confirmatory versus disconfirmatory outcomes, yet did not change our overall conclusions (see the Supplementary Materials). Future studies should more directly explore the role of a choice-confirmation bias mechanism in the context of latent updating of unchosen actions using dedicated designs.

Another relevant theory for our findings is “divisive normalization,” suggesting a neural mechanism by which only relative, but not absolute, values of options are maintained (*51*, *58*–*60*). Following this line of thought, value update for the chosen action should affect (i.e., “normalize”) the values of other alternatives that were not selected. However, the divisive normalization theory makes no specific distinction between two types of unchosen options—options that were offered but not selected and options that were unavailable in a specific trial. Such a distinction seems necessary to explain our findings. According to the divisive normalization theory, both unchosen-offered and unavailable options should be subjected to a “normalizing” value change based on the value update of the chosen option. Here, we showed the influence of monetary outcome on the preference of offered-unchosen options when contrasted with the previously unavailable options, which is not accounted for by divisive normalization. Thus, divisive normalization in its current form could not account for our findings. Recently, Fouragnan and colleagues (*61*) conducted ground-breaking research in macaques showing that the value of a temporarily unavailable option was distinctly maintained by hippocampal activity, while activity in the medial orbitofrontal cortex/ventromedial prefrontal cortex was important for comparing the values of available options. Speculatively, this may suggest that the value of the offered but unchosen alternative has increased activity compared to unoffered alternatives during value updating. While our work shows a distinct and latent value update of unchosen-offered options, further work is needed to determine whether unavailable options were also updated to a lesser extent or completely shielded from value update.

Our results should be discussed in relation to the “choice-induced preference change” effect. This phenomenon has been studied extensively in the field of psychology, and it suggests that the act of making a choice itself can influence our preferences and attitudes toward options. Specifically, studies have shown that regardless of the outcome, when deliberating upon two options, the mere act of choice leads to a “spreading of alternatives.” Therefore, under choice-induced preference theory, the act of making a choice itself leads to increased value for the chosen option, while the value of an unchosen option decreases (*62*–*67*). Choice-induced preference effects are independent of observed outcomes and can occur in the absence of an outcome. However, the current study was specifically designed to estimate outcome-dependent credit assignment to unchosen actions. Hence, we demonstrated both reduced and increased value of actions that were unchosen based on the value of the delivered outcome. Moreover, while choice-induced change is tightly related to the deliberation process, the current effect is not moderated by deliberation properties. Thus, our results cannot be explained by choice-induced preference alone or seen as providing evidence in favor or against such a theoretical notion. Note that we did find deliberation effects that were independent of reward (see Fig. 2). Specifically, we observed that more difficult choices and longer decisions were associated with a reduced inclination to choose the previously unselected card (see the Supplementary Materials), which can be seen as evidence for choice-induced preference change of unchosen actions. Further studies should directly explore whether latent credit assignment to unchosen actions and choice-induced preference bear any mechanistic associations.

Our findings are also consistent with a limited luck theory, which suggests that humans may perceive luck as a limited resource (*53*, *68*). This theory proposes that individuals may assume that obtaining a reward from one option implies that the other option is less lucky and therefore less likely to generate a reward. This tendency may reflect a basic property of ecological environments, where resources are limited and often correlated (*69*). This has been suggested to lead to “zero-sum thinking” according to which a gain for one option entails a loss for the other (*70*). In our computerized experiment, rewards were abundant and uncorrelated by design, so our conclusions may be limited to this particular design (*71*). To address this limitation, we emphasized the lack of correlation between action values in our replication study. However, it could be insufficient to override preexisting assumptions that humans have about the way in which rewards are distributed. Previous studies have shown individuals to perceive a negative correlation between the value of two offered options even when they could physically see that the pool of associated outcomes for the two options was separated and independent (*53*). Further research should examine whether the updating of the unchosen action’s value is modulated by the true value dependencies of the environment and by individual experiences.

Another important and relevant mechanism is reference point dependency (*72*). According to this notion, individuals update action values not only based on an observed outcome of the selected action but also based on a mental value reference point that is updated across all actions. Specifically, Palminteri and Lebreton have shown that an objective outcome is mentally weighted by a mental reference, thus leading to a phenomenon where an action with a higher true expected value could be chosen less compared with an action with lower true expected values due to differences in the underlying reference point (i.e., also termed state value in the reinforcement learning literature). However, we argue that the effect reported in the current study could not be predicted by a reference point dependence mechanism. To illustrate this, consider that the individual was offered cards A and B and that A was chosen and followed by some observed outcome. Now, let us further consider that in the next trial, B (the unchosen arm) is reoffered with a different card (e.g., C or D). We found in the current study strong and replicated evidence suggesting that if A was rewarded, B is now less likely to be selected in a preceding “B vs. C” trial. In the same sense, if A was unrewarded, B is now more likely to be selected in the next B versus C trial. The reference point dependence mechanism cannot explain such an effect since the observed outcome following the selection of A would be only assigned to update the reference value of A versus B. Without making further A versus B choices, an update of a reference point has no effect on the actions’ subjective value and should not influence the choices in the next B versus C trial. However, our evidence clearly shows such an association. This should not be taken as evidence against or in favor of the notion that individuals might weigh the observed outcome against a reference point when making value-based decisions. It is simply that the phenomenon of inverse credit assignment for unchosen actions is not predicted by such mechanisms, which makes it less relevant to the current study.

Our findings are relevant in the context of an anticipated regret minimization theory (*73*), which proposes that individuals make decisions not only to maximize their rewards but also to minimize their future regret (*5*). Moreover, when the outcome of foregone alternatives is not observable, counterfactual thinking theory (*6*) suggests that people tend to engage in “what if …” thinking patterns, envisioning the potential outcomes of their unselected actions. Further studies are needed to explore whether the select-reject mechanism proposed in the current study and specifically the speculated maintenance of reject values correspond to regret and counterfactual “what if …” processes in humans.

Our findings are also in line with previous neural studies that used full feedback (i.e., the outcomes of both the chosen and unchosen options were shown to the subjects) and suggested the existence of two distinct prediction-error signals, one for the actual reward and one for the foregone outcome (*19*–*22*). Specifically, these studies recorded dopamine striatal levels (known to encode reward prediction error) with high temporal resolution in humans performing a gambling task (*19*). Dopamine fluctuations in the striatum were found to encode a combination of reward prediction errors, including counterfactual prediction errors defined by the unobtained outcome of unchosen options. A recent study demonstrated that when individuals were able to reject a certain option but were subsequently shown the outcome of the foregone alternative, striatal activity represented a prediction error with a reversed sign (*74*). Further neuroimaging studies are needed to confirm the existence of a counterfactual prediction error when the outcome of the rejected action remains hidden from the observer. Another unique perspective on the described value updating phenomenon is provided by the reinforcement learning theory of dopamine function, specifically the opponent actor learning model (*75*). This influential computational model is inspired by findings about the distinct role of the dopaminergic D1 and D2 neural pathways in approach and avoidance learning, respectively (*76*–*78*). Further studies should explore whether our findings correspond with the same pathways. Namely, our model argues that each choice is a weighted sum of selecting an action and rejecting an alternative. Further studies should explore whether selecting/rejecting an action involves similar neural mechanisms to the ones described in approach-avoid literature (*79*).

To conclude, we extended previous findings suggesting latent and inversed value updating for unchosen actions by demonstrating it in a sequential decision-making task. Furthermore, we found evidence contradicting the hypothesis that this type of updating is driven by the act of deliberation, which was suggested to negatively bind the chosen and unchosen actions in memory. Alternatively, we suggest a select-reject value learning model to best explain this phenomenon, which is aligned with emerging neurological descriptions of the dual reward prediction-error system.

## MATERIALS AND METHODS

### Participants

One hundred seventy-eight prolific workers (age mean, 26.1; range, 18 to 51; 101 males, 76 females, and 1 other) completed an online experiment in return for monetary compensation. All participants reported normal or corrected vision and no current or past psychiatric or neurological diagnosis. The study protocol was approved by the Research Ethics Council of Tel-Aviv University, and all participants signed informed consent before participating in the study.

### Reinforcement learning task

Participants completed an online multi-armed bandit reinforcement learning task where they were asked to choose cards to gain monetary rewards. The task included four cards, and in each trial, the computer randomly selected and offered two for participants to choose from. Each card led to a reward according to an expected value that drifted across the trials [generated using a random walk with a noise of *N*(0,0.03)]. The task included two conditions (win versus loss block) manipulated between four interleaved blocks (whether the first block was win or loss was counterbalanced between participants). In a “win” block, the only possible outcomes were winning 1 or 0 play dollars, and in the “loss” condition, the only possible outcomes were losing 0 or 1 play dollar (henceforward addressed as rewarded and unrewarded, respectively, for convenience). These two conditions allowed us to test whether inverse value updating for unchosen actions is more pronounced under win versus loss blocks, thus achieving evidence related to former findings, which found outcome-context–specific effects (*53*). At the start of the session, participants were presented with task instructions, completed a short practice, and completed a multichoice quiz, which they had to complete with 100% accuracy. Participants were told that they needed to do their best to earn as much money as possible. To make sure that the reported effects are not due to some misunderstanding leading participants to wrongly assume that the cards’ expected values are dependent, we report a replication study where both instructions and the quiz emphasized that finding a reward under one card had no meaning to how good/bad other cards were (see the Supplementary Materials). Participants completed four blocks, with 50 trials each, and at the end of the experiment were paid a fixed amount (£2.5) plus a bonus (of £1 or £1.5) based on their performance. Further information and trial sequence is described in Fig. 1 and the Supplementary Materials.

### Data treatment

In the first trial on each block, trials with implausibly quick RTs (<200 ms) or exceptionally slow RTs (>4000 ms) were omitted (1.79% of all trials). Participants with more than 10% excluded trials (21 participants) or higher than 5% no-response rate (4 participants), in total 25 participants (12.3% of subjects; age mean, 22.8; range, 18 to 36; 22 males, 3 females), were excluded altogether. To conduct our main behavioral analysis, we selected a subset of trials in which the previously unoffered card was reoffered, and the previously offered card was not. This resulted in an average of 63.6 trials per participant (SD = 6.7), with the number of trials ranging from 46 to 81 across subjects.

### Bayesian parameter estimation

Bayesian logistic regression and reinforcement learning computational modeling analyses were performed using “brms,” “rstan,” and “loo” packages in R (*39*, *40*, *80*). All models included population-level (fixed effects) and participant-level (random effects) parameters for all estimated models and were sampled with weakly informative priors. All chains were visually examined using trace plots, pairs plots, and R-hat estimates and were found to show good convergence. We report the median, HDI_95%_, and probability of direction for parameters’ posterior distributions (for logistic regression, estimates are on the log-odds scale; for prior robustness checks, see the Supplementary Materials) (*81*). For computational models, parameter estimation was conducted using the rstan package in R, and model comparisons were made using the leave-one-block-out approach and the loo package in R (*40*, *82*). For each model, we held out one of the four blocks and then calculated the elpd for each trial in the held-out block. This was repeated for all blocks, resulting in a matrix of 4000 MCMC samples × 34,096 observations. We then used the elpd R function to obtain a pointwise estimation for each observation and the loo_compare function (from the loo package) to perform pairwise model comparisons between each model and the model with the largest elpd. An elpd difference of two times the SE was considered substantial (*40*).

### Simulation of data based on each models’ empirical population parameters

We simulated 175 agents for each of the four computational models to examine the model’s ability to produce the main behavioral effects observed in the empirical data (see Fig. 4). To gain an empirical estimate of population parameters, we first fitted a hierarchical Bayesian reinforcement learning model to the empirically observed behavior. This allowed us to gain population-level parameter posterior estimates for each parameter of each model. We sampled parameters for each agent from the empirical posterior distribution and simulated artificial choice data using the same task design as was used to collect empirical human data (e.g., number of arms, true expected values, and number of blocks and trials). This allowed us to obtain artificial data for each model based on empirical population parameters, which we then used to estimate the existence of the regression signature we found in empirical data (see Fig. 4 and the Supplementary Materials).

### Parameter recovery

To establish the suitability of each of our three models, we tested our ability to recover simulated parameters (*38*). For that purpose, we first simulated data from each of the three generative models. Specifically, 175 agents were simulated hierarchically, separately for each of the three different models (i.e., double updating with two prediction errors, double updating with one prediction error, and select-reject models). For our simulation, we set population-level parameter values as follows: (i) model 1—double updating with two prediction errors: ɑ_ch_ = 0.3, ɑ_unch_ = 0.1, ꞵ = 4; (ii) model 2—double updating with one prediction error: ɑ_ch_ = 0.3, ɑ_unch_ = 0.1, ꞵ = 4; and (iii) model 3—select-reject: ɑ = 0.3, ω = 0.7, ꞵ = 4. Individual-level parameters were then sampled from a normal distribution with a mean defined by the population-level parameter (fixed effect) and an SD of 1 for ɑ and ω, or 1.5 for ꞵ. The ɑ and ω parameters were scaled to be between 0 and 1 using a logit transformation. To accommodate extreme cases where ɑ_ch_ < ɑ_unch_, we truncated *Q* values in the rare event that they exceeded the range of [0,1]. For every agent, four blocks containing 50 trials each were simulated using the same task used to collect empirical data. We used weakly informative priors [population location parameters ~*N*(0,2), population-scale parameters ~Cauchy (0,2), and individual-level parameters ~*N*(0,1)] and sampled from the posterior distribution using the rstan package in R (*39*). Specifically, we used 20 chains with 1000 warm-up and 50 sampling iterations each. We examined chain convergence using trace plots and Rhat estimates (*83*). We then examined the match between true and recovered parameters, both at the population and individual levels. Figure 3 illustrates the results for each of the three parameters for every model. Specifically, we found excellent recoverability for population-level (fixed effects) parameters with all true parameters being well within the HDI_95%_ of the updated posteriors. We further found good parameter recovery at the individual level, as can be seen by high correlations between the individual true and recovered parameters (all Pearson estimates are above 0.75; all *P* values < 0.01; see Fig. 3). Thus, we were able to recover with high accuracy and precision all simulated parameter values both at the population and at the individual levels (see Fig. 3).

### Model recovery

We used a model recovery method to further ensure our ability to distinguish latent reinforcement learning mechanisms from empirical data. We simulated data from each generative model (i.e., double updating with two prediction errors, double updating with one prediction error, and select-reject models; see the “Parameter recovery” section for simulation parameter specifications). For each of the three artificial datasets, we then calculated an elpd separately for each of the four models (baseline, double updating with two prediction errors, double updating with one prediction error, and select-reject models). Elpd was calculated using the same leave-one-block-out method used for model comparison in the empirical data analysis. Specifically, we trained each model while holding one block out (as a test set). We then calculated the elpd for each agent, trial, and posterior sample in the test block. This was repeated with all blocks, allowing us to gain an elpd estimate across all blocks. Each training used 20 MCMC chains with 1000 warm-up and 50 sampling iterations, which produced 1000 posterior samples. We then used the loo R package to compare elpd across models. We used a widely accepted thumb rule where a difference of more than 2 SDs in elpd estimates can be considered substantial (*40*, *82*). We found excellent results, with all three models being adequately recovered using our method (Table 1).

## Supplementary Materials

### This PDF file includes:

Supplementary Text Figs. S1 to S5 Tables S1 and S2 References
