# Supplementary material for: Computational mechanisms underlying latent value updating of unchosen actions
Source: Sci Adv. 2023 Oct 20;9(42):eadi2704. doi: 10.1126/sciadv.adi2704 (PMC10588947; doi:10.1126/sciadv.adi2704)
Supplement: Supplementary file 1 — Supplementary Text Figs. S1 to S5 Tables S1 and S2 References [file sciadv.adi2704_sm.pdf]

Science Advances
AAAS

Supplementary Materials for

Computational mechanisms underlying latent value updating of unchosen actions

Ido Ben-Artzi *et al.*

Corresponding author: Ido Ben-Artzi, idobenartzi@mail.tau.ac.il

Sci. Adv. **9**, eadi2704 (2023)
DOI: 10.1126/sciadv.adi2704

This PDF file includes:

Supplementary Text
Figs. S1 to S5
Tables S1 and S2
References

Supplementary Text

Multi-armed bandit task

Each trial started with a one-second fixation point followed by the presentation of two offered cards (abstract fractal images; see Figure 1). The two cards were randomly selected out of a deck of four cards and were randomly assigned to one of the two sides of the screen (i.e., left and right; Figure 1). Participants choose a card using a right/left corresponding response-key press ('s' or 'k' keys in a QWERTY keyboard; until response with a 6sec deadline). After the choice was made, a choice feedback screen with only the chosen card appeared (500 ms), followed by an outcome screen which also introduced the monetary outcome (£0 or £1 for win blocks, and -£1 or £0 for loss blocks) presented in the middle of the screen along with the selected card (1000 ms). The task included four blocks of 50 trials, two of each outcome condition (win vs. loss), in an interleaved order. Whether the first block was a win or loss was counterbalanced between participants (win-loss-win-loss or loss-win-loss-win). Every block started with an instruction screen that introduced the subject with four new cards to be used in that block and the two related outcomes (+£1/0 for win blocks or £0/-1 for loss blocks). Win blocks, in which the participant could either gain one game-coin or no-coin, were indicated by a green frame that appeared across the screen edges constantly across the whole block. Loss blocks, in which the outcome could either be a loss of a game-coin or no-coin, were indicated by a similar red frame.

Hierarchical Bayesian logistic regression for model-independent analysis

We performed hierarchical Bayesian logistic regression in R using 'brms' package (*80*). We used weakly informative priors of N(0,0.2) for all fixed effects. Both prior and posterior predictive checks confirmed that these priors were adequate to the prediction of our dependent variables (i.e., select previously unchosen card). To make sure our results are not heavily influenced by our priors, we also conducted a prior sensitivity analysis. We found that the previous-outcome (rewarded vs. unrewarded) had a substantial influence on the probability of selecting in trial n+1 the previously unchosen option also when using narrower (N(0,0.09) (posterior median =-0.21, $HDI_{95\%}$ between -0.29 and -0.14; probability of direction = 100%) or wider (N(0,0.4) priors (posterior median =-0.25, $HDI_{95\%}$ between -0.33 and -0.18; probability of direction = 100%).

Replication of the effect in a different dataset

During the instruction phase of the task reported in the main text, we did not stress explicitly that the expected values of the different cards are independent. We, therefore, wanted to demonstrate that the effect reported in the main text (influence of previous-outcome on the probability of selecting an unchosen option) was not due to some misunderstanding during the instruction phase, which led some participants to implicitly assume that an outcome for a certain card bears some meaning regarding the other cards. For this reason, we re-analyzed a data set from our lab where participants performed a multi-armed reinforcement learning task, similar to the one reported in the main text (N=49 undergraduate psychology students from Tel Aviv University, mean age = 23.6, SD = 5.82; 43 women, 7 men (*23*)). However, here, during the instructions phase, participants saw a screen that was dedicated to explicitly explaining that the presented outcomes are associated only with the bandit that was chosen and had no meaning to the other unselected ones. Specifically, the cover story of the current task postulated that the task included four cars, two offered to the individual each trial, and participants were asked to choose one which they think has a better chance to drive without causing an accident. During the

instructions, participants were explicitly told that "learning about one car's accident chances has nothing to do with the other cars' accident chances", and that more than one car could be a safe/dangerous car at any given trial. The trial sequence was similar to the one in the main text. The only difference was that unrelated to the specific current analysis, participants in this specific task were also presented with threat/arousal cues at trial initiation (neutral, threat, or no visual cue). We then performed a logistic hierarchical Bayesian regression, examining the influence of previous-outcome (unrewarded vs. rewarded) on the chance of selecting a car that was offered and unchosen at trial *n*, at the next trial *n*+1. We further examined only trials where the chosen car at trial *n* was not reoffered at trial *n*+1. We found clear evidence suggesting that participants tended to choose the previously unchosen card less often in trial *n*+1 if trial n was rewarded (45%) vs. unrewarded (51%; posterior median =-0.19, $HDI_{95\%}$ between -0.32 and -0.06; probability of direction = 99.8%).

Examining each model's ability to generate the main behavioral effects.

We sought to estimate the same regression signatures we reported in the main text for each model using the simulated data (see Figure 4 in the main text). We therefore fitted a Bayesian hierarchical logistic regression where previous-choice predicted the selection of previously unchosen actions (only for trials where the previously chosen action was not reoffered, exactly as we did with the empirical data analysis, see main text). For the baseline model, we found a fixed effect in the opposite direction of the empirical effect (see Figure 4; posterior median = .05, $HDI_{95\%}$ between -.02 and .12, probability of direction = 90%). All the other three models showed the empirical behavioral signature (Double updating with two prediction-errors: posterior median = -.07, $HDI_{95\%}$ between -.14 and. 04, probability of direction = 97%; Double updating with one prediction-error: posterior median=-.07, $HDI_{95\%}$ between -.15 and -.00, probability of direction = 98%; Select-reject: posterior median=-.16, $HDI_{95\%}$ between -.23 and -.09, probability of direction = ~100%; see Figure 4 in the main text). Finally, we estimated the moderation of delta-EV on the updating of unchosen action in each simulated data set. We repeated the same regression mentioned above only with delta-EV and the paired interaction with previous-outcome as additional predictors. We found that when using the reward function from our task all models were able to reproduce the main effect of delta-EV as in the empirical data (see Figure 4, main text).

Optimistic bias

In response to a reviewer's concern, we wanted to examine whether a differential update for the chosen action for rewarded / unrewarded outcomes could give rise to our behavioral effect even in the absence of any update for unchosen actions (Figure 1C). Specifically, previous studies suggested an "optimistic bias" mechanism where the update of the chosen action following a rewarded outcome is bigger than the update that follows an unrewarded outcome (*84*, *85*). We simulated 175 agents using an optimistic updating model which was identical to the baseline model (Eq. 1-3, main text) with the only difference that two learning rates were included as free parameters; α_{ch+} for positive prediction errors and α_{ch-} for negative prediction-error. The agents' simulated parameters were sampled from $\alpha_{ch+} \sim N(.3,1)$, $\alpha_{ch-} \sim N(.1,1)$ and $\beta \sim N(4,1.5)$. We fitted a Bayesian hierarchical logistic regression model to the simulated data where previous-outcome predicted the selection of an unchosen action in the next trial (see "Influence of reward on unchosen actions" section in the main text). Unlike the regression fitted to empirical data, here we found no evidence in favor of an effect (median posterior = .03; $HDI_{95\%}$ between -.04 and

.11; see Figure S4. Therefore, an optimistic bias for the update of chosen action is unlikely to be the cause for the effect we observed in empirical data.

Choice-confirmation bias

Previous studies suggested a choice-confirmation bias mechanism where both chosen and unchosen actions show larger value updating following choice-confirming compared to disconfirming outcomes (*43*, *54–57*). Specifically, previous studies asked participants to complete a reinforcement learning task under conditions of full feedback where both the outcome obtained for the chosen action and the foregone outcome associated with the unchosen action were presented (*43*, *56*). These studies found evidence for a larger value update for chosen actions after positive compared to negative obtained outcomes. Furthermore, a larger value update for unchosen actions was found after negative compared with positive foregone outcomes. Hence, Palminteri & Lebreton (2022) concluded that participants tend to show a greater update for outcomes that confirm their choice. In the current study, participants were presented only with the feedback associated with the chosen action and did not observe any feedback for the forgone action. To provide a preliminary examination of the choice-confirmation bias mechanism in the current choice data, we further examined an extension of the three computational models that were examined in the main text (double updating with one prediction error, double updating with two prediction errors, and select-reject model) to include a choice-confirmatory mechanism. Specifically, we extended the single learning rate in each model to include two learning-rate free parameters, one for rewarded outcomes (α_{CON}**)** and one for unrewarded outcomes (α_{DIS}). We then tested using leave-one-block-out cross-validation the ability of each model to predict an unseen block. First, following previous studies, we found a higher learning rate for confirmatory vs. disconfirmatory outcomes across all choice-confirmatory extensions (see Table S2). Second, we found that the select-reject model had a better fit compared with the two alternative models (yet to a smaller extent compared to the difference found in the main text for the second-best fitting model; Δelpd<2SE; (*40*)). While the current finding did not change our overall conclusion, further dedicated studies and paradigms are required to explore in-depth the choice-confirmation bias mechanism in the context of value update of latent unchosen actions.

Association between choice-difficulty and reaction-times

In the main text, we sought to examine whether choice-difficulty (estimated using absolute delta expected-value; $|\Delta EV|$) and reaction-times (RT) moderate the main effect where the value of unchosen actions is updated based on the feedback given for the chosen action. Previous studies have indicated that higher choice-difficulty should result in longer RTs. Here, we wanted to perform a sanity check that will assure that this well-familiar pattern exists in our data, while also making sure that the correlation is not too high to the point where the two measures of deliberation are completely dependent. Therefore, we performed a hierarchical Bayesian regression analysis where RTs were predicted using absolute delta expected-value ($|\Delta EV|$). As expected, we found a strong and negative association so that for every 0.1 unit increase in delta expected-value, the model predicted a -15.3ms change in RTs ($HDI_{95\%}$ between -20.4 and -10.3; R^2 of .24, R^2 $HDI_{95\%}$ between .23 to .26; see Figure S5).

Table S1. Descriptive summary of empirical parameters for each of the four computational models

**Baseline model**	
α	.30 [.23 to .37]
β	2.0 [1.8 to 2.3]
**Double updating with two prediction-errors**	
α_{chosen}	.26 [.2 to .32]
$\alpha_{unchosen}$	.04 [.02 to .06]
β	2.2 [2.0 to 2.5]
**Double updating with one prediction-error**	
α_{chosen}	.31 [.24 to .37]
$\alpha_{unchosen}$	.05 [.03 to .07]
β	1.9 [1.6 to 2.1]
**Select-reject value learning**	
α	.37 [.29 to .43]
ω	.78 [.73 to .84]
β	2.2 [2.0 to 2.5]

Note. Estimates are median of marginal posterior population distributions. Brackets provide 95% CI.

Table S2. Empirical parameter estimates for choice-confirmation bias models

**Model**	α_{CON}	α_{DIS}	elpd
Single PE confirmatory	.32 (.27 to .37)	.08 (.05 to .11)	-22053 (58.4)
Double PE confirmatory	.38 (.34 to .43)	.10 (.07 to .13)	-21822 (59.9)
Select-reject confirmatory	.36 (.31 to .43)	.14 (.07 to .24)	-21805 (61)

Note. The table provides the median posterior estimation for each learning rate, followed by the $HDI_{95\%}$ *in brackets.*

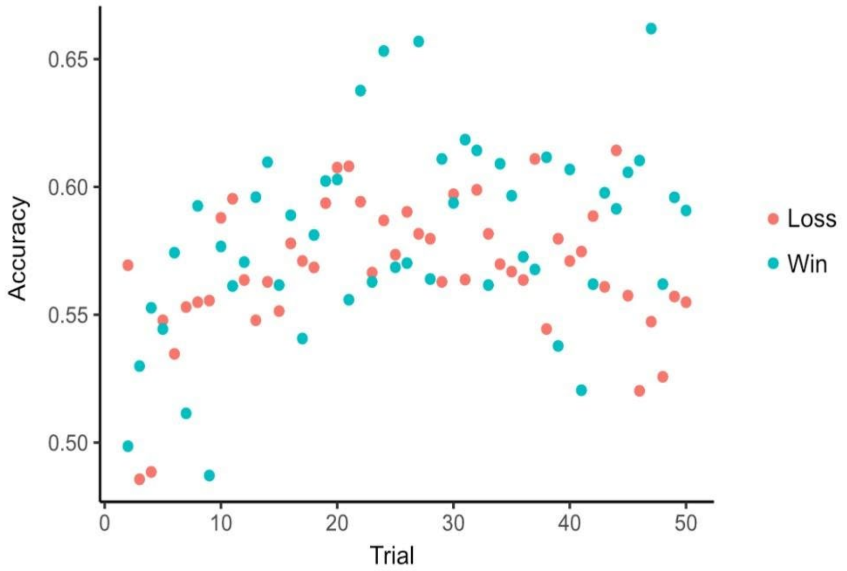

Fig. S1. Accuracy rate as a function of trial progression. For each trial, we defined an accurate choice as a selection of the card with the higher true expected value between the two offered cards. Overall, this plot indicates that participants learned the value of the cards and were able to perform the task well.

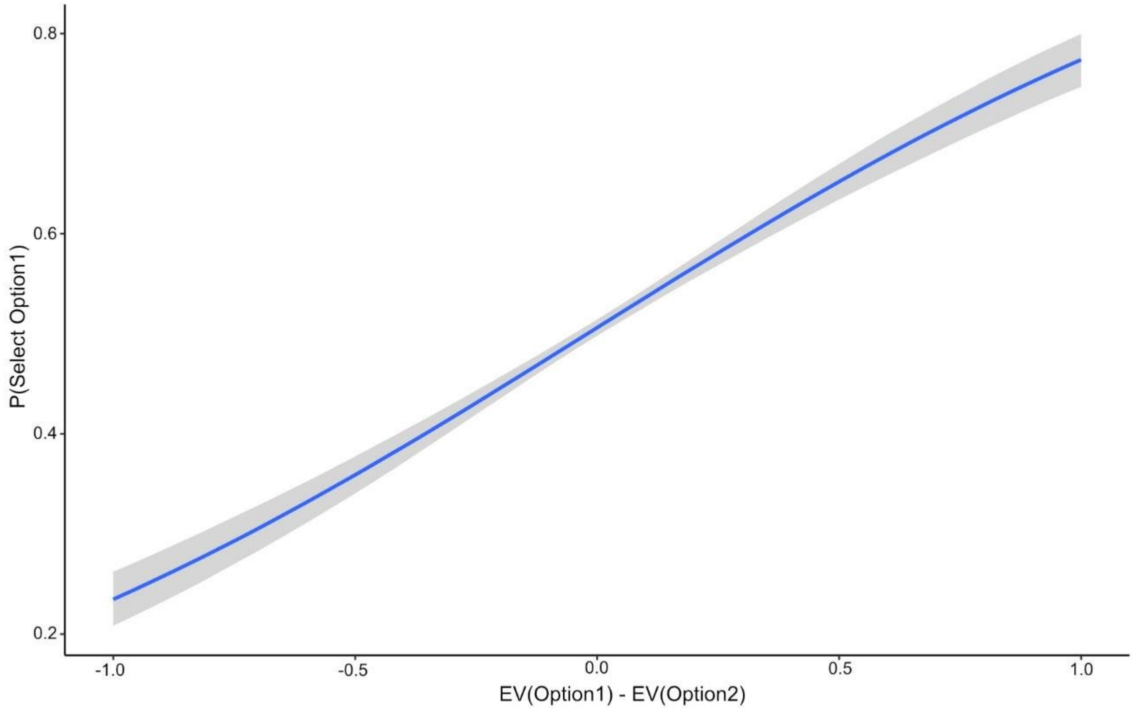

Fig. S2. Participants choice was modulated by latent expected values. To Ensure participants had learned the true expected values of the cards, we ran a hierarchical Bayesian logistic regression. Specifically, we predicted participants' choices by the difference in expected value of the two cards. We found robust evidence showing participants were more likely to choose a certain option when its value was higher (posterior median = 1.2, $HDI_{95\%}$ between 1.05 and 1.35; probability of direction = ~100%). Thus, we conclude participants indeed learned the latent expected values of the cards.

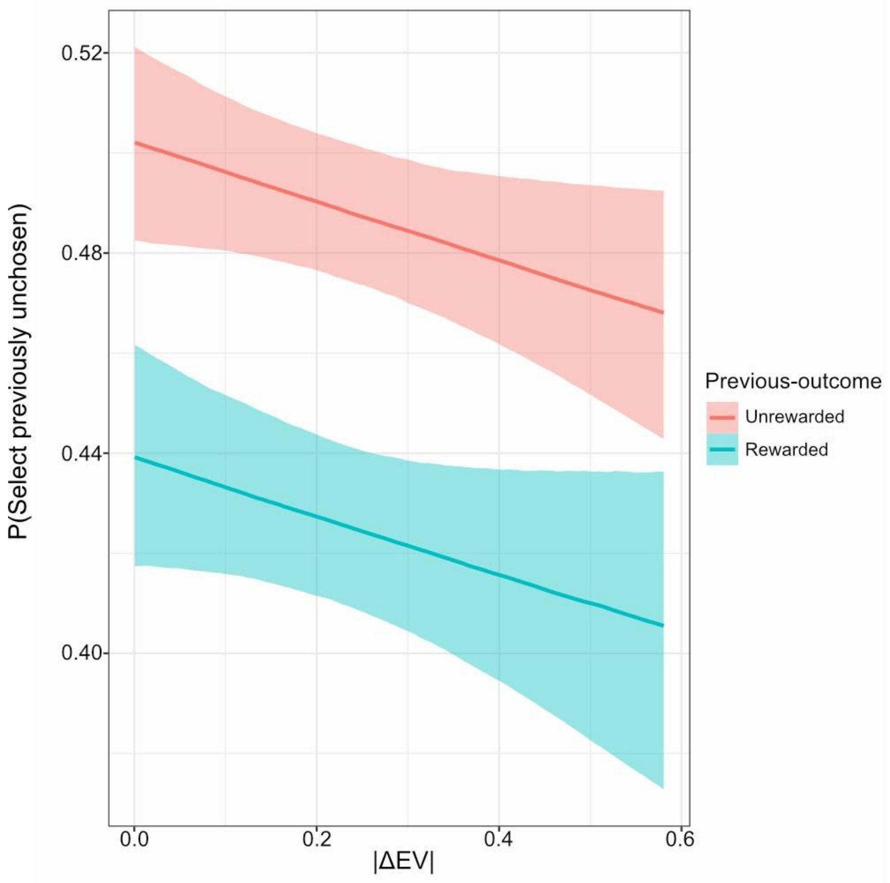

Fig. S3. Inverse value update of unchosen actions is independent of the arms' current value. We wanted to ensure participants change their preference for the previously unchosen card depending on the reward obtained, irrespectively of the true values of the cards offered in trial n+1. We, therefore, added the absolute difference in expected values in trial n+1 to our main regression model. This plot illustrates the effect remains for all levels of delta EV. Overall, we found inverse value update of unchosen actions to occur independently of the difference in the expected value of the offered option.

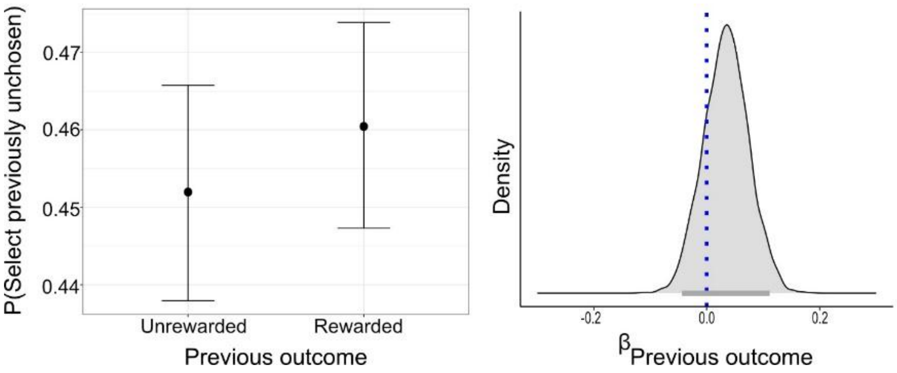

Fig. S4. The effect of previous-outcome on the selection of unchosen actions in data simulated from an optimistic bias model. (A) The effect of previous-outcome on the selection of unchosen actions in data simulated from an optimistic bias model. **(B)** The posterior distribution for the effect of previous-outcome on the selection of unchosen action (the gray line shows $HDI_{95\%}$ and the blue line shows the posterior median). Overall, we found that an optimistic bias does not produce the regression effect observed in empirical data and reported in Figure 1C.

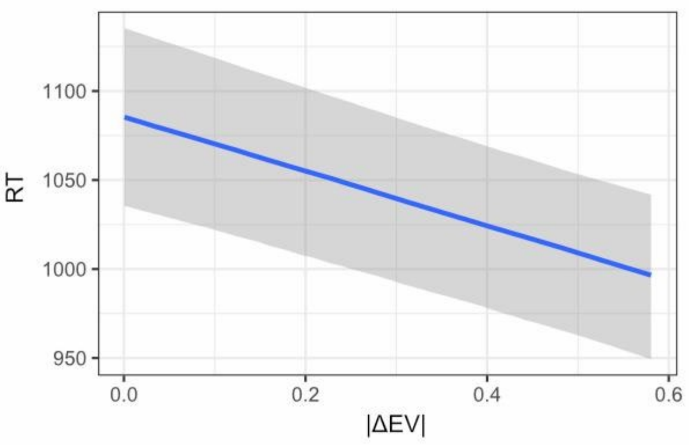

Fig. S5. Examining the relationship between reaction times and choice-difficulty. RTs and choice-difficulty are known in the literature to be highly correlated. We therefore wanted to assert that these two measures which we used as separate properties of the deliberation process are indeed mutually independent. For this purpose, we executed a simple linear regression analysis where RTs were predicted by choice difficulty (absolute difference in expected values between the two arms). As expected, we found a strong and negative association.

REFERENCES AND NOTES

1. R. S. Sutton, A. G. Barto, *Introduction to Reinforcement Learning* (MIT Press, 1998), vol. 135.

2. S. Wang, S. F. Feng, A. M. Bornstein, Mixing memory and desire: How memory reactivation supports deliberative decision-making. *Wiley Interdiscip. Rev. Cogn. Sci.* **13**, e1581 (2022).

3. A. Tversky, I. Simonson, Context-dependent preferences. *Manage. Sci.* **39**, 1179–1189 (1993).

4. D. L. Schacter, R. G. Benoit, K. K. Szpunar, Episodic future thinking: Mechanisms and functions. *Curr. Opin. Behav. Sci.* **17**, 41–50 (2017).

5. D. E. Bell, Regret in decision making under uncertainty. *Oper. Res.* **30**, 961–981 (1982).

6. N. J. Roese, Counterfactual thinking. *Psychol. Bull.* **121**, 133–148 (1997).

7. T. Connolly, M. Zeelenberg, Regret in decision making. *Curr. Dir. Psychol. Sci.* **11**, 212–216 (2002).

8. A. M. Bornstein, K. A. Norman, Reinstated episodic context guides sampling-based decisions for reward. *Nat. Neurosci.* **20**, 997–1003 (2017).

9. A. M. Bornstein, M. W. Khaw, D. Shohamy, N. D. Daw, Reminders of past choices bias decisions for reward in humans. *Nat. Commun.* **8**, 15958 (2017).

10. W. J. Brogden, Sensory pre-conditioning. *J. Exp. Psychol.* **25**, 323–332 (1939).

11. G. E. Wimmer, D. Shohamy, Preference by association: How memory mechanisms in the hippocampus bias decisions. *Science* **338**, 270–273 (2012).

12. J. A. Dusek, H. Eichenbaum, The hippocampus and memory for orderly stimulus relations. *Proc. Natl. Acad. Sci. U.S.A.* **94**, 7109–7114 (1997).

13. N. W. Schuck, Y. Niv, Sequential replay of non-spatial task states in the human hippocampus. *Science* **364**, eaaw5181 (2019).

14. D. Shohamy, A. D. Wagner, Integrating memories in the human brain: Hippocampal–midbrain encoding of overlapping events. *Neuron* **60**, 378–389 (2008).

15. B. P. Staresina, L. Davachi, Mind the gap: Binding experiences across space and time in the human hippocampus. *Neuron* **63**, 267–276 (2009).

16. S. Bavard, S. Palminteri, Why unchosen options linger in our minds. *Commun. Biol.* **4**, 1271 (2021).

17. N. Biderman, D. Shohamy, Memory and decision making interact to shape the value of unchosen options. *Nat. Commun.* **12**, 4648 (2021).

18. J. R. Busemeyer, J. T. Townsend, Decision field theory: A dynamic-cognitive approach to decision making in an uncertain environment. *Psychol. Rev.* **100**, 432–459 (1993).

19. K. T. Kishida, I. Saez, T. Lohrenz, M. R. Witcher, A. W. Laxton, S. B. Tatter, J. P. White, T. L. Ellis, P. E. M. Phillips, P. R. Montague, Subsecond dopamine fluctuations in human striatum encode superposed error signals about actual and counterfactual reward. *Proc. Natl. Acad. Sci. U.S.A.* **113**, 200–205 (2016).

20. T. Lohrenz, K. McCabe, C. F. Camerer, P. R. Montague, Neural signature of fictive learning signals in a sequential investment task. *Proc. Natl. Acad. Sci. U.S.A.* **104**, 9493–9498 (2007).

21. M. J. Tobia, R. Guo, U. Schwarze, W. Boehmer, J. Gläscher, B. Finckh, A. Marschner, C. Büchel, K. Obermayer, T. Sommer, Neural systems for choice and valuation with counterfactual learning signals. *Neuroimage* **89**, 57–69 (2014).

22. B. Y. Hayden, J. M. Pearson, M. L. Platt, Fictive reward signals in the anterior cingulate cortex. *Science* **324**, 948–950 (2009).

23. Y. Troudart, N. Shahar, Formation of non-veridical action-outcome associations following exposure to threat-related cues. *Emotion* **23**, 2094–2099 (2023).

24. N. Biderman, S. J. Gershman, D. Shohamy, The role of memory in counterfactual valuation. *J. Exp. Psychol. Gen.* **152**, 1754–1767 (2023).

25. R. Ratcliff, A theory of memory retrieval. *Psychol. Rev.* **85**, 59–108 (1978).

26. R. Ratcliff, J. N. Rouder, Modeling response times for two-choice decisions. *Psychol. Sci.* **9**, 347–356 (1998).

27. R. Ratcliff, G. McKoon, The diffusion decision model: Theory and data for two-choice decision tasks. *Neural Comput.* **20**, 873–922 (2008).

28. G. Wright, P. Ayton, Decision time, subjective probability, and task difficulty. *Mem. Cognit.* **16**, 176–185 (1988).

29. T. Hanks, R. Kiani, M. N. Shadlen, A neural mechanism of speed-accuracy tradeoff in macaque area LIP. *eLife* **3**, e02260 (2014).

30. P. D. Kvam, Modeling accuracy, response time, and bias in continuous orientation judgments. *J. Exp. Psychol. Hum. Percept. Perform.* **45**, 301–318 (2019).

31. U. Maoz, G. Yaffe, C. Koch, L. Mudrik, Neural precursors of decisions that matter—An ERP study of deliberate and arbitrary choice. *eLife* **8**, e39787 (2019).

32. G. M. Stine, E. M. Trautmann, D. Jeurissen, M. N. Shadlen, A neural mechanism for terminating decisions. *Neuron*, **111**, 2601–2613 (2023).

33. N. A. Ruiz, S. DuBrow, V. P. Murty, Agency as a bridge to form associative memories. *J. Exp. Psychol. Gen.* **152**, 1797–1813 (2023).

34. H. M. Bayer, P. W. Glimcher, Midbrain dopamine neurons encode a quantitative reward prediction error signal. *Neuron* **47**, 129–141 (2005).

35. W. Schultz, P. Dayan, P. R. Montague, A neural substrate of prediction and reward. *Science* **275**, 1593–1599 (1997).

36. V. Agrawal, P. Shenoy, Tracking what matters: A decision-variable account of human behavior in bandit tasks. *Proc. Annu. Meet. Cogn. Sci. Soc.* **43** (2021)

37. S. Palminteri, V. Wyart, E. Koechlin, The importance of falsification in computational cognitive modeling. *Trends Cogn. Sci.* **21**, 425–433 (2017).

38. R. C. Wilson, A. G. Collins, Ten simple rules for the computational modeling of behavioral data. *eLife* **8**, e49547 (2019).

39. B. Carpenter, A. Gelman, M. D. Hoffman, D. Lee, B. Goodrich, M. Betancourt, M. Brubaker, J. Guo, P. Li, A. Riddell, Stan: A probabilistic programming language. *J. Stat. Softw.* **76**, 1 (2017).

40. A. Vehtari, A. Gelman, J. Gabry, Practical Bayesian model evaluation using leave-one-out cross-validation and WAIC. *Stat. Comput.* **27**, 1413–1432 (2017).

41. A. Vehtari, D. P. Simpson, Y. Yao, A. Gelman, Limitations of Bayesian leave-one-out cross-validation for model selection. *Comput. Brain Behav.* **2**, 22–27 (2019.

42. E. D. Boorman, T. E. Behrens, M. F. Rushworth, Counterfactual choice and learning in a neural network centered on human lateral frontopolar cortex. *PLOS Biol.* **9**, e1001093 (2011).

43. S. Palminteri, G. Lefebvre, E. J. Kilford, S.-J. Blakemore, Confirmation bias in human reinforcement learning: Evidence from counterfactual feedback processing. *PLOS Comput. Biol.* **13**, e1005684 (2017).

44. L. A. Henkel, M. Mather, Memory attributions for choices: How beliefs shape our memories. *J. Mem. Lang.* **57**, 163–176 (2007).

45. M. Lind, M. Visentini, T. Mäntylä, F. Del Missier, Choice-supportive misremembering: A new taxonomy and review. *Front. Psychol.* **8**, 2062 (2017).

46. R. P. Heitz, The speed-accuracy tradeoff: History, physiology, methodology, and behavior. *Front. Neurosci.* **8**, 150 (2014).

47. A. Furstenberg, H. Sompolinsky, L. Y. Deouell, Error monitoring when no errors are possible: Arbitrary free-choice decisions invoke error monitoring processes. *iScience* **26**, 106373 (2023).

48. A. Furstenberg, A. Breska, H. Sompolinsky, L. Y. Deouell, Evidence of change of intention in picking situations. *J. Cogn. Neurosci.* **27**, 2133–2146 (2015).

49. E. Ullmann-Margalit, S. Morgenbesser, Picking and choosing. *Soc. Res.* **44**, 757–785 (1977).

50. D. Katsimpokis, G. E. Hawkins, L. van Maanen, Not all speed-accuracy trade-off manipulations have the same psychological effect. *Comput. Brain Behav.* **3**, 252–268 (2020).

51. T. A. Klein, M. Ullsperger, G. Jocham, Learning relative values in the striatum induces violations of normative decision making. *Nat. Commun.* **8**, 16033 (2017).

52. J. Li, N. D. Daw, Signals in human striatum are appropriate for policy update rather than value prediction. *J. Neurosci.* **31**, 5504–5511 (2011).

53. D. Marciano, E. Krispin, S. Bourgeois-Gironde, L. Y. Deouell, Limited resources or limited luck? Why people perceive an illusory negative correlation between the outcomes of choice options despite unequivocal evidence for independence. *Judgm. Decis. Mak.* **14**, 573–590 (2019).

54. V. Chambon, H. Théro, M. Vidal, H. Vandendriessche, P. Haggard, S. Palminteri, Information about action outcomes differentially affects learning from self-determined versus imposed choices. *Nat. Hum. Behav.* **4**, 1067–1079 (2020).

55. M. Lebreton, K. Bacily, S. Palminteri, J. B. Engelmann, Contextual influence on confidence judgments in human reinforcement learning. *PLOS Comput. Biol.* **15**, e1006973 (2019).

56. S. Palminteri, M. Lebreton, The computational roots of positivity and confirmation biases in reinforcement learning. *Trends Cogn. Sci.* **26**, 607–621 (2022).

57. T. Schüller, A. G. Fischer, T. O. J. Gruendler, J. C. Baldermann, D. Huys, M. Ullsperger, J. Kuhn, Decreased transfer of value to action in Tourette syndrome. *Cortex* **126**, 39–48 (2020).

58. S. Gluth, N. Kern, M. Kortmann, C. L. Vitali, Value-based attention but not divisive normalization influences decisions with multiple alternatives. *Nat. Hum. Behav.* **4**, 634–645 (2020).

59. K. Louie, L. E. Grattan, P. W. Glimcher, Reward value-based gain control: Divisive normalization in parietal cortex. *J. Neurosci.* **31**, 10627–10639 (2011).

60. K. Louie, M. W. Khaw, P. W. Glimcher, Normalization is a general neural mechanism for context-dependent decision making. *Proc. Natl. Acad. Sci. U.S.A.* **110**, 6139–6144 (2013).

61. E. F. Fouragnan, B. K. H. Chau, D. Folloni, N. Kolling, L. Verhagen, M. Klein-Flügge, L. Tankelevitch, G. K. Papageorgiou, J.-F. Aubry, J. Sallet, M. F. S. Rushworth, The macaque anterior cingulate cortex translates counterfactual choice value into actual behavioral change. *Nat. Neurosci.* **22**, 797–808 (2019).

62. D. J. Bem, Self-perception: An alternative interpretation of cognitive dissonance phenomena. *Psychol. Rev.* **74**, 183–200 (1967).

63. L. Festinger, *Conflict, Decision, and Dissonance* (Stanford Univ. Press, 1964).

64. K. Voigt, C. Murawski, S. Bode, Endogenous formation of preferences: Choices systematically change willingness-to-pay for goods. *J. Exp. Psychol. Learn. Mem. Cogn.* **43**, 1872–1882 (2017).

65. M. K. Chen, J. L. Risen, How choice affects and reflects preferences: Revisiting the free-choice paradigm. *J. Pers. Soc. Psychol.* **99**, 573–594 (2010).

66. T. Sharot, C. M. Velasquez, R. J. Dolan, Do decisions shape preference? Evidence from blind choice *Psychol. Sci.* **21**, 1231–1235 (2010).

67. T. Sharot, S. M. Fleming, X. Yu, R. Koster, R. J. Dolan, Is choice-induced preference change long lasting? *Psychol. Sci.* **23**, 1123–1129 (2012).

68. D. Marciano-Romm, A. Romm, S. Bourgeois-Gironde, L. Y. Deouell, The alternative omen effect: Illusory negative correlation between the outcomes of choice options. *Cognition* **146**, 324–338 (2016).

69. G. M. Foster, Peasant society and the image of limited good. *Am. Anthropol.* **67**, 293–315 (1965).

70. S. Davidai, S. J. Tepper, The psychology of zero-sum beliefs. *Nat. Rev. Psychol.* **2**, 472–482 (2023).

71. E. Schulz, N. T. Franklin, S. J. Gershman, Finding structure in multi-armed bandits. *Cogn. Psychol.* **119**, 101261 (2020).

72. S. Palminteri, M. Lebreton, Context-dependent outcome encoding in human reinforcement learning. *Curr. Opin. Behav. Sci.* **41**, 144–151 (2021).

73. M. Zeelenberg, Anticipated regret, expected feedback and behavioral decision making. *J. Behav. Decis. Mak.* **12**, 93–106 (1999).

74. G. Jocham, T. A. Klein, M. Ullsperger, Differential modulation of reinforcement learning by D2 dopamine and NMDA glutamate receptor antagonism. *J. Neurosci.* **34**, 13151–13162 (2014).

75. A. G. E. Collins, M. J. Frank, Opponent actor learning (OpAL): Modeling interactive effects of striatal dopamine on reinforcement learning and choice incentive. *Psychol. Rev.* **121**, 337–366 (2014).

76. A. V. Kravitz, L. D. Tye, A. C. Kreitzer, Distinct roles for direct and indirect pathway striatal neurons in reinforcement. *Nat. Neurosci.* **15**, 816–818 (2012).

77. M. J. Frank, Dynamic dopamine modulation in the basal ganglia: A neurocomputational account of cognitive deficits in medicated and nonmedicated parkinsonism. *J. Cogn. Neurosci.* **17**, 51–72 (2005).

78. M. J. Frank, K. Hutchison, Genetic contributions to avoidance-based decisions: Striatal D2 receptor polymorphisms. *Neuroscience* **164**, 131–140 (2009).

79. M. J. Frank, L. C. Seeberger, R. C. O'Reilly, By carrot or by stick: Cognitive reinforcement learning in parkinsonism. *Science* **306**, 1940–1943 (2004).

80. P.-C. Bürkner, Advanced Bayesian multilevel modeling with the r package brms. *R. J.* **10**, 395–411 (2018).

81. D. Makowski, M. S. Ben-Shachar, S. H. A. Chen, D. Lüdecke, Indices of effect existence and significance in the Bayesian framework. *Front. Psychol.* **10**, 2767 (2019).

82. J. Piironen, A. Vehtari, Comparison of Bayesian predictive methods for model selection. *Stat. Comput.* **27**, 711–735 (2017).

83. P.-C. Bürkner, M. Scholz, S. T. Radev, Some models are useful, but how do we know which ones? Towards a unified Bayesian model taxonomy. arXiv:2209.02439 [stat.ME] (6 September 2022).

84. G. Lefebvre, M. Lebreton, F. Meyniel, S. Bourgeois-Gironde, S. Palminteri, Behavioural and neural characterization of optimistic reinforcement learning. *Nat. Hum. Behav.* **1**, 1–9 (2017).

85. C.-C. Ting, S. Palminteri, M. Lebreton, J. B. Engelmann, The elusive effects of incidental anxiety on reinforcement-learning. *J. Exp. Psychol. Learn. Mem. Cogn.* **48**, 619–642 (2022).
